# French guidelines for the etiological workup of eosinophilia and the management of hypereosinophilic syndromes

**DOI:** 10.1186/s13023-023-02696-4

**Published:** 2023-04-30

**Authors:** Matthieu Groh, Julien Rohmer, Nicolas Etienne, Wadih Abou Chahla, Antoine Baudet, Aurélie Chan Hew Wai, Cécile Chenivesse, Irena Clisson Rusek, Vincent Cottin, Matthieu Decamp, Pascal De Groote, Fanny Delahousse, Nicolas Duployez, Stanislas Faguer, Frédéric Gottrand, Florent Huang, Thierry Leblanc, Antoine Magnan, Thierry Martin, Geoffrey Mortuaire, Antoine Néel, Luc Paris, Arnaud Petit, Julien Rossignol, Nicolas Schleinitz, Juliette Soret-Dulphy, Delphine Staumont-Salle, Benjamin Terrier, Louis Terriou, Jean-François Viallard, Guillaume Lefèvre, Jean-Emmanuel Kahn

**Affiliations:** 1 Department of Internal Medicine, National Reference Center for Hypereosinophilic Syndromes (CEREO), Hôpital Foch, 40, Rue Worth, 92151 Suresnes, France; 2 Department of Internal Medicine, Hôpital Foch, Suresnes, France; 3 Inserm, U1286 - INFINITE - Institute for Translational Research in Inflammation, University of Lille, CHU Lille, Lille, France; 4 Department of Internal Medicine, University of Sorbonne-Paris-Cité, APHP, CHU Bichat, Paris, France; 5 Department of Infectious Diseases and Tropical Medicine, University of Sorbonne-Paris-Cité, APHP, CHU Necker-Enfants Malades, Paris, France; 6 Department of Pediatric Hematology, University of Lille, CHU Lille, Lille, France; 7 Department of Internal Medicine, CH Annecy Genevois, Metz Tessy, France; 8 Department of Pharmacology, Hôpital Foch, Suresnes, France; 9 CNRS, Inserm, Institut Pasteur de Lille, U1019-UMR9017-CIIL-Centre d’Infection et d’Immunité de Lille, University of Lille, CHU Lille, Lille, France; 10 CRISALIS (Clinical Research Initiative in Severe Asthma: a Lever for Innovation and Science), F-CRIN Network, INSERM US015, Toulouse, France; 11 Association Pour l’Information sur les Maladies à Eosinophiles, Bourg-la-Reine, France; 12 Department of Respiratory Medicine, Hôpital Louis Pradel, UMR754 INRAE, University of Lyon 1, Hospices Civils de Lyon, Lyon, France; 13 Department of Cytogenetics, CHU de Caen, Caen, France; 14 Department of Cardiology, University of Lille, CHU Lille, Lille, France; 15 Nantes, France; 16 Laboratory of Hematology, University of Lille, CHU Lille, Lille, France; 17 CNRS, Inserm, IRCL, UMR9020 - UMR1277 - Canther - Cancer Heterogeneity, Plasticity and Resistance to Therapies, 59000 Lille, France; 18 Department of Nephrology and Organ Transplantation, University of Paul Sabatier Toulouse III, CHU Toulouse, Toulouse, France; 19 Division of Gastroenterology, Hepatology and Nutrition, Department of Paediatrics, Jeanne de Flandre Children’s Hospital, University of Lille, CHU Lille, Lille, France; 20 Department of Cardiology, Hôpital Foch, Suresnes, France; 21 Pediatric Hematology and Immunology Department, University Sorbonne-Paris-Cité, APHP, CHU Robert Debré, Paris, France; 22 Department of Respiratory Medicine, Hôpital Foch, Suresnes, France; 23 Department of Internal Medicine, CHU Strasbourg, Strasbourg, France; 24 Otorhinolaryngology-Head and Neck Department, University of Lille, CHU de Lille, Lille, France; 25 Department of Internal Medicine, CHU Nantes, Nantes, France; 26 Department of Parasitology and Mycology, Sorbonne Université, APHP, CHU Pitié-Salpêtrière, Paris, France; 27 Department of Hematology and Pediatric Oncology, Sorbonne Université, APHP, CHU Armand Trousseau, Paris, France; 28 Department of Hematology, University of Sorbonne-Paris-Cité, APHP, CHU Necker, Paris, France; 29 Department of Internal Medicine, APHM, CHU La Timone, Marseille, France; 30 Centre d’Investigation Clinique, University of Sorbonne-Paris-Cité, AP-HP, CHU St-Louis, Paris, France; 31 Department of Dermatology, University of Lille, CHU de Lille, Lille, France; 32 Department of Internal Medicine, University of Sorbonne-Paris-Cité, AP-HP, Paris, France; 33 Department of Internal Medicine and Clinical Immunology, University of Lille, CHU de Lille, Lille, France; 34 Department of Internal Medicine, CHU de Bordeaux, Bordeaux, France; 35 Institut d’Immunologie, University of Lille, CHU de Lille, Lille, France; 36 Department of Internal Medicine, University of Paris Saclay, APHP, CHU Ambroise Paré, Boulogne-Billancourt, France

**Keywords:** Hypereosinophilia, Hypereosinophilic syndromes, Recommendation, Management

## Abstract

Eosinophilic-related clinical manifestations are protean and the underlying conditions underpinning eosinophilia are highly diverse. The etiological workup of unexplained eosinophilia/hypereosinophilia can be challenging, and can lead sometimes to extensive, inappropriate, costly and/or invasive investigations. To date, guidelines for the etiological workup and management of eosinophilia are mainly issued by hematologists, and thus mostly cover the scope of clonal hypereosinophilic syndromes (HES). Here, thanks to an extensive literature review, and thanks to the joint work of a large panel of experts involving physicians from both adult and pediatric medicine and from various subspecialties (as well as a representative of a patients’ association representative), we provide recommendations for both the step-by step diagnostic workup of eosinophilia (whether unexplained or within specific contexts) as well as the management and follow-up of the full spectrum of eosinophilic disorders (including clonal, reactive, lymphocytic and idiopathic HES, as well as single-organ diseases). Didactic prescription summaries intended to facilitate the prescription of eosinophil-targeted drugs are also provided, as are practical diagnostic and therapeutic algorithms. Lastly, this set of recommendations also includes a summary intended for general practitioners, as well as an overview of the therapeutic patient education program set up by the French reference center for HES. Further updates will be mandatory as new validated information emerges.

**Supplementary Information:**

The online version contains supplementary material available at 10.1186/s13023-023-02696-4.

## Aims

The aim of these recommendations established by national experts is to inform and assist health professionals in the diagnostic and/or therapeutic management of patients with hypereosinophilic syndrome (HES), including organ-specific eosinophilic disorders (excluding eosinophilic esophagitis, for which there are dedicated guidelines, and hypereosinophilic asthma).

It is intended as a practical guide that attending physicians and non-expert physicians can refer to for the initial diagnostic workup and/or follow-up of patients with hypereosinophilia (HE)/HES. It is also intended to optimize and harmonize the management and follow-up of HE/HES. A summary of this set of recommendations dedicated for General Practitioners is available as Additional file 1: Appendix 1.

This document is the product of a multidisciplinary effort involving physicians (from both adult and pediatric medicine) from various specialties (Cytogenetics, Gastroenterology, Hematology, General Medicine, Infectious Diseases, Internal Medicine, ENT, Pediatrics, Pharmacy, Pulmonology, Dermatology, Cardiology, Nephrology, Intensive Care Medicine) as well as a patient association’s representative.

Due to the wide range of clinical conditions grouped under HES, the complexity and diversity of eosinophil-associated disorders, this expert consensus is not a substitute for expert opinion on a case-by-case basis. It does, however, set out the basics of the management of HE/HES patients, and will be updated as new, validated information emerges.

## Methodology

This set of recommendations was prepared according to the "Method for designing and drafting a rare disease National Diagnostic and Care Protocol " published by the French National Authority for Health (2012). After a critical review and compilation of data from the international literature, the writing group proceeded to prepare a first draft in accordance with the relevant framework, which was then submitted to a multidisciplinary review group. The corrected document was then reviewed and approved by the multidisciplinary group. Full details of the methodology are available at https://has-sante.fr/upload/docs/application/pdf/2022-06/pnds_she_argumentaire.pdf.

## General information

### Definitions

#### Eosinophilia, HE and HES

The classification of eosinophilic disorders currently used for patient management and research is that proposed in 2011 by the International Cooperative Working Group on Eosinophil Disorders (ICOG-EO) which has recently been revised. Above all, this classification distinguishes blood eosinophilia (between 0.5 and 1.5 × 10^9^/L eosinophils), HE (blood eosinophilia > 1.5 × 10^9^/L and/or tissue eosinophilia) and HES [1, 2]. Detailed criteria for each condition are listed in Box 1.

**Box 1:** Definition of eosinophilia, HE and HES (adapted from Valent et al. J Allergy Clin Immunol [1]; and Valent et al. Allergy [3])

Condition	Definition
Blood eosinophilia	Eosinophil count between 0.5 and 1.5 × 10^9^/L eosinophils
Hypereosinophilia (HE)	Eosinophil count > 1.5 × 10^9^/L on two occasions one month apart and/or tissue eosinophilia (as defined in “Definition of HES-related organ involvement” section)
Hypereosinophilic syndrome (HES)	Blood HE AND
	Organ damage or dysfunction caused by tissue eosinophils (as defined in “Definition of HES-related organ involvement” Section), AND
	Exclusion of other possible causes of organ dysfunction
HES/organ-specific eosinophilic disease	Blood and/or tissue eosinophilia AND
	Single-organ/tract involvement

The two key concepts of this classification are:

- On the one hand, the need to rule out causes of organ dysfunction other than eosinophilic infiltration.
- On the other hand, the distinction between asymptomatic HE and HES, which implies that clinical organ dysfunction is presumably due to eosinophil toxicity.

This distinction has very practical consequences for patient management: hence, the diagnosis of HES is made as soon as blood and/or tissue HE is found to be the cause of clinical organ dysfunction (regardless of the underlying mechanism/etiology of HE). While this definition is broad, it is a useful reminder that organ dysfunction associated with a high eosinophil count and eosinophil toxicity can occur regardless of the cause of HE (e.g., including in parasitic infections) [3]. In this setting, both an etiological workup for HE as well as onset of treatment for HE should be considered without delay.

Finally, it is important to note that in case of functional or even life-threatening impairment requiring urgent onset of treatment, a minimal delay of 1 month is no longer required before the diagnosis of HES can be retained.

#### Various causes of HE

The various causes of HE/HES are usually classified according to their underlying pathophysiological mechanisms. Thus, four major distinct clinical case definitions are usually distinguished:

##### Clonal HE (previously myeloid variant of HE/HES)

Clonal HE is caused by a hematopoietic neoplasm associated with abnormal proliferation of eosinophilic precursors. In this setting, all clonal eosinophil granulocytes carry the same cytogenetic or molecular abnormality. This is, for example, the case of chronic eosinophilic leukemia associated with the 4q12 deletion (resulting in the loss of the *CHIC2* gene) responsible for the fusion of the *FIP1L1* and *PDGFRA* genes (F/P + chronic eosinophilic leukemia (or myeloid neoplasm with F/P-associated HES according to the most recent WHO and International Consensus Classification nomenclatures) [4–6]. As other molecular abnormalities have been identified, failure to detect the *FIP1L1::PDGFRA* fusion gene does not necessarily rule out the diagnosis of clonal HE [7, 8].

##### Reactive HE

Reactive eosinophilia encompasses all conditions (allergies, autoimmune diseases, solid or hematological malignancies etc.), leading to the abnormal production of high amounts of eosinophilopoietic cytokines—including interleukin (IL)-5—and thus to the polyclonal expansion of eosinophils and subsequent organ damage [1–3]. Of note, lymphocytic-variant HE/HES, an indolent T cell lymphoproliferative disorder in which abnormal T cells produce high amounts of IL-5, is also classified as reactive HE [9–11].

##### HE of undetermined significance (HEus)

This is a subtype of HE in which both the etiological and organ impact assessments are negative. The diagnosis remains provisional and may change if eosinophil-related clinical manifestations occur during follow-up. Nevertheless, HEus may persist on the long-term, sometimes with very high eosinophil counts (e.g. > 5.0 × 10^9^/L), yet without any clinical manifestation [12].

##### Familial HE

Multiple cases of unexplained HE found within the same family. Due to the rarity of the reported cases, there is no simple explanation for familial forms, but some studies have targeted the 5q31-5q33 region (genes encoding IL-3, IL-5, GM-CSF) [13, 14].

#### Definition of HES-related organ involvement

In the current classifications, HE-related organ involvement is defined by:

- At least one of the following clinical criteria:
  - Fibrosis (cardiac, digestive tract, skin, etc.).
  - Venous or arterial thrombosis.
  - Involvement of the skin or mucosa (pruritus, eczema, prurigo, urticaria, angioedema, ulceration, purpura).
  - Central or peripheral nervous system involvement.
  - Pulmonary manifestations.
  - Gastrointestinal involvement.
  - Eosinophilic vasculitis.
  - Clinical involvement of other organs (liver, kidneys, pancreas, etc.) may be present but is less common and subject to evidence that organ damage indeed is related to eosinophilia.
- Histological or cytological criteria:
  - Eosinophilic infiltration of the bone marrow > 20% and/or
  - Eosinophilic infiltration of tissue deemed excessive by the pathologist and/or
  - Presence of extracellular deposition of eosinophil cationic protein (ECP), major basic protein (MBP) and eosinophil peroxidase (EPX) on immunohistochemistry.

By extension, if there is no evidence of eosinophilic tissue infiltration (e.g., high-risk biopsy procedure, pre-existing treatment with systemic corticosteroids, etc.), HES-related organ involvement can also be diagnosed when all the following criteria are present: 1. Blood HE; 2. organ dysfunction compatible with HES; 3. parallel course of organ dysfunction and blood HE.

Although there is no strict correlation between blood and tissue eosinophilia, blood eosinophilia tends to be a sound surrogate of tissue eosinophilia. Besides specific situations (e.g. acute pulmonary eosinophilia at the acute phase [15], some patients with eosinophilic myocarditis) [16], the presence of clinical manifestations despite normal AEC is uncommon in HES and rather suggests disease sequela or an alternative diagnosis. Hence, in the latter situation, tissue biopsies seeking for persistent tissue eosinophilia should be considered before the diagnosis of HES flare is retained.

The main clinical manifestations of HES are listed in Box 2. In an international case series of 188 patients (including all disease subtypes), the most common symptoms were cutaneous (69%), respiratory (44%), gastrointestinal (38%) and cardiac (20%) [17].

**Box 2:** Main clinical manifestations of HES

*Cutaneous*: pruritus, eczema, urticaria, angioedema, bullae, ulceration of the limbs or mucous membranes, splinter hemorrhages, fasciitis, livedo, purpura.
Pulmonary: asthma, bronchiolitis, bronchitis, bronchiectasis, interstitial lung disease.
*Cardiac*: myocarditis, pericarditis, valvular disease, endomyocardial fibrosis, dilated cardiomyopathy, intracavitary thrombus, coronary artery vasospasm.
*Neurological*: ischemic cerebrovascular disease (usually bilateral and of watershed distribution), peripheral neuropathy, myelitis.
*Digestive*: eosinophilic esophagitis, eosinophilic gastritis, eosinophilic enteritis and eosinophilic colitis, eosinophilic ascites, eosinophilic cholangitis.
Arterial and/or venous thrombosis.
*Rheumatologic*: arthritis, tenosynovitis, myositis.
Thromboangiitis obliterans–like vasculopathy or eosinophilic vasculitis (excluding EGPA or PAN).

Hypereosinophilic asthma with sinonasal polyposis are common manifestations of T2 diseases, usually restricted to airways in most patients but can also be part of HES.

### Epidemiology

HES is a rare disorder for which robust epidemiologic data are scarce. Also, the diversity of the conditions that fall within the spectrum of HES makes it difficult to accurately capture the incidence and prevalence of HES and associated eosinophilic disorders. Based on the North American Surveillance, Epidemiology and End Results cancer registry and the International Classification of Diseases for Oncology, the mean annual incidence of HES between 2001 and 2005 was estimated to be 0.36 new cases per year per million inhabitants. However, this study used only registry data and did not provide details of various HES subtypes [18].

In contrast, in a national retrospective study of F/P + chronic eosinophilic leukemia conducted in collaboration with all public and private laboratories testing for F/P in France, the number of F/P + patients identified between 2003 and 2018 was 195, thereby corresponding to an average incidence of 0.18 new cases per year per million inhabitants [19].

Lastly, in more general terms, relatively similar rates of patients with F/P + chronic eosinophilic leukemia or lymphocytic HES were reported in the main case series of HES published to date, each in the range of 5–10% [20]. As there are about 200 patients with F/P rearrangement in France, the number of patients with HES (all types) in France is roughly estimated to be between 2000 and 4000.

### Etiological factors

#### Eosinophil differentiation and homeostasis

Eosinophils are produced in the bone marrow from the differentiation of hematopoietic stem cell progenitors. Their differentiation, maturation and release into the peripheral blood is orchestrated by a specific combination of transcription and growth factors, the most important being IL-5, IL-3 and GM-CSF. These cytokines can be produced by TH2-polarized CD4+ T cells, type 2 innate lymphoid cells (ILC2s), mast cells and mesenchymal cells, as well as eosinophils themselves [21, 22].

Under physiological conditions, moderate eosinophilic infiltration may be found mainly in the gastrointestinal (GI) tract (except for the esophagus), with an eosinophil count generally less than 30–60 per microscopic high‐powered field (HPF), depending on the stage [23]. In the physiological state, the recruitment of blood eosinophils to these tissues is dependent on eotaxin and other chemotactic mediators.

In pathological conditions, abnormal eosinophilic tissue infiltration may be found in these same or other organs (skin, bronchi, myocardium, etc.). In inflammatory state, the increase in chemotactic factors in the affected organ likely explains the tissue recruitment of eosinophils.

Eosinophils store numerous cationic proteins in their granules, such as matrix basic protein, eosinophil cationic protein, eosinophil peroxydase and eosinophil-derived neurotoxin, as well as cytokines (including IL-3, 5, 6 and 13; TNF-alpha; and TGF-beta) and lipid mediators with cytotoxic and/or procoagulant effects [24–28]. Depending on the receptors present on their surfaces and the microenvironmental signals detected, eosinophils can release the contents of their granules selectively and/or produce newly formed mediators that can be toxic within infiltrated tissue, but also amplify (or conversely, regulate) the local immune response. Eosinophils are also involved in tissue remodeling, which partly explains the fibrotic lesions (affecting the skin, the heart) that can be observed in patients with HES [29].

#### Genetic factors

Data regarding genetic factors predisposing to or responsible for HES are scarce, and no genome-wide association study has yet been performed. A *JAK1* gain-of-function germline mutation was recently identified in a mother and her two sons whose clinical symptoms were consistent with multi-refractory systemic HES and who all responded to targeted treatment with a JAK inhibitor [30]. Finally, a noncoding germline variant creating a gain-of-function enhancer upregulating IL-5 transcription was recently identified in a family with five generations of familial HE/HES [31].

#### Environmental factors

Factors that trigger exacerbations of HES are suspected in patients with a relapsing–remitting disease course, e.g., dietary triggers in gastroduodenal or colonic HES. However, there are currently no data to support the role of these environmental factors in either the occurrence or the recurrence of HE.

### Clinical course and long-term prognosis

Nowadays, given the current state of knowledge and available treatments, only clonal forms and severe cardiac involvement are truly life-threatening [32].

The dramatic improvement of the survival of clonal HES patients is largely attributable to imatinib which is highly effective in all disease subtypes associated with genes fusions involving *PDGFRA* or *PDGFRB* (which used to be considered as the most severe disease subtypes) [19, 33]. In this line, in a French case series of 151 patients with F/P + chronic eosinophilic leukemia treated with imatinib, the 1-, 5- and 10-year survival rates were 100%, 98% and 89% [19].

In chronic eosinophilic leukemia not associated with *PDGFRA* or *PDGFRB*, the prognosis is related to the risk of progression to acute myeloid leukemia [34, 35]. Similarly, both *FGFR1* and *FLT3*-related HES are aggressive hematologic malignancies, which have a poor prognosis in the absence of bone marrow allograft transplantation [7, 8].

Conversely, the main risk of lymphocytic HES is the progression to high-grade peripheral T-cell lymphoma, estimated to be around 5–10% of patients within series [36].

Finally, the prognosis of idiopathic HES depends mainly on the severity of the initial complications, with cardiac, CNS and thrombotic symptoms being the most severe HES manifestations [37–41]. In other cases, the prognosis of idiopathic HES patients is most likely close to that of the general population, with corticosteroids showing remarkable efficacy in severe complications and the use of steroid-sparing treatments, including new biologic therapies, likely to revolutionize the management of non-clonal HES.

### Treatment

**Fig. 1:**
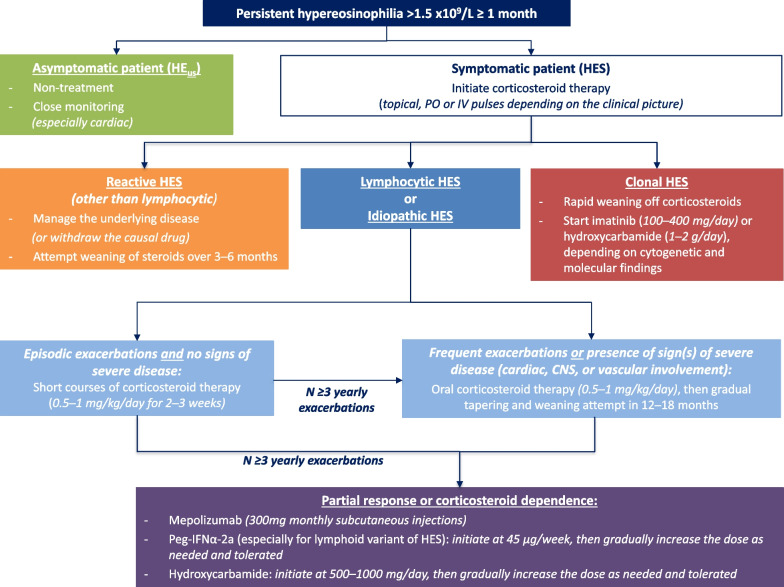
Proposed algorithm for the therapeutic management of patients treated for HE or HES

Generally, the treatment depends on the type of HES, its severity (cardiac, CNS or thrombotic involvement in particular), its clinical course (continuous progression or relapsing/remitting course) and the patient's specific background (age, possible comorbidities) [20, 42]. Its duration is variable. Treatment can be either sporadic (in the case of occasional non-severe exacerbations) or prolonged (in the case of frequent exacerbations or if signs of severity are present from the outset). The goal is generally to control the clinical symptoms and achieve normalization of the eosinophil count. However, HE may be tolerated in some cases if the patient undergoes regular clinical and laboratory monitoring for potential complications (including cardiac involvement). A general algorithm for the management of HES is provided in Fig. 1. The use of tyrosine kinase inhibitors has radically transformed the prognosis of patients with clonal HES, while biologic therapies targeting the IL-5 pathway may reduce the morbidity associated with prolonged systemic corticosteroids.

## Initial assessment

### Aims

In the early management of HE, the main goals are to:

- Identify the underlying cause of HE/HES using a comprehensive individual diagnostic approach.
- Identify major eosinophil-related organ involvements and assess their severity.
- Rule out differential diagnoses (not all types of organ dysfunctions associated with HE are necessarily due to HE).
- Establish therapeutic indications, considering any comorbidities likely to affect the prognosis or treatment tolerability.
- Arrange follow-up with the attending physician and relevant specialists.

### Professionals involved

Due to the protean manifestations of HE/HES, any physician is likely to be confronted with HE, either incidentally during a routine laboratory workup or HE-related organ involvement (HES).

Hence, HE/HES should initially be managed by:

- A general practitioner or pediatrician who will conduct a history-taking interview and perform a comprehensive physical examination (possibly supplemented by basic first-line investigations) to screen for the most common causes of HE (drug-related or parasitic infection, as detailed in “Pharmacological treatments” section) and potential related complications. This initial workup must not delay the referral to an organ specialist physician if there are any unusual symptoms or if serious (cardiac, respiratory, thrombotic, neurological) disease is suspected.
- Given the wide range of associated disorders, many (pediatric and adult) specialists are involved in the management of patients with HE/HES: internists, hematologists, pulmonologists, dermatologists, gastroenterologists, cardiologists, ENT specialists, allergologists, rheumatologists, and urologists.

The follow-up of patients with HE/HES can generally be coordinated by the referring physician, regardless of their specialty, who must, however, ensure that all patients are regularly screened for various possible organ complications (through questioning and routine comprehensive physical examinations, as well as specific additional tests), which may sometimes involve organs other than those initially affected during follow-up.

If necessary, advice can be sought from a tertiary referral center for HES (the list of French referral centers is detailed in Additional file 2: Appendix 2 and available at www.cereo.fr).

### Initial diagnostic approach to HE

#### General principles

The first basic elements from the medical history that are used to guide the etiological workup are the duration of HE, the level of blood eosinophilia (< or > 1.5 × 10^9^/L) and potential HE-related symptoms [43]. Generally, there is no correlation between the level of blood HE and the severity of clinical manifestations (which is more closely related to the extent of eosinophilic tissue infiltration and the degree of eosinophil activation within tissues).

#### Main causes to be considered in the presence of eosinophilia between 0.5 and 1.5 × 10^9^/L

The main conditions to be considered are atopy and parasitic infections that do not require a tissue cycle. The main parasitic infections in metropolitan France are pinworm, tapeworm, and scabies. In patients with a history of previous travels to parasite-endemic areas, strongyloidiasis should also be considered. Other less common causes include Addison's disease and HIV infection. In daily practice, in the presence of obvious atopic disease (asthma, allergic rhino-conjunctivitis, atopic dermatitis), it is acceptable to restrict further investigations in the absence of any other finding suggestive of neither eosinophil-related organ involvement repercussions nor another than atopy.

#### Main causes to be considered in the presence of HE > 1.5 × 10^9^/L

##### In the presence of recent onset HE > 1.5 × 10^9^/L: etiologic investigations should focus on drugs and parasites

The investigation of a drug etiology tends to be challenging, and causality is often difficult to establish. The duration of HE (and the temporal relationship between its onset and the initiation of a drug, typically 2–8 weeks) is an important consideration. Likewise, the history of previous CBC can be instructive. The main drug classes that are routinely implicated in HE are non-steroidal anti-inflammatory drugs, antiepileptic drugs, antibiotics, sulfonamides and allopurinol [37, 44]. More recently, dupilumab (an anti-IL4/IL13 biologic) and immune checkpoint inhibitors have also been shown to provide HE [45, 46]. Overall, all medicinal products have the potential to induce HE, as do iodinated contrast media, herbal remedies, dietary supplements, as well as some dialysis membranes (Box 3). Iatrogenic eosinophilia typically presents as a skin rash, but other clinical pictures (pulmonary, cardiac, gastrointestinal, etc.) without skin involvement are possible, as is also isolated asymptomatic HE. Noteworthy, skin manifestations may be absent in DRESS (drug reaction with eosinophilia and systemic symptoms), whereas signs of lymphocyte activation (mononucleosis syndrome) are possible in this setting. The RegiSCAR score and viral PCR tests (EBV, CMV and HHV-6) may also be useful [47]. Nevertheless, evidence of viral replication is neither necessary nor sufficient to make a diagnosis of DRESS and should solely be included within the entire clinical picture.

**Box 3:** Non-exhaustive list of HE-inducing drugs

*Antibiotics*: penicillins, cephalosporins, cyclins (mainly minocycline), sulfonamides, nitrofurantoin, isoniazid, rifampicin, vancomycin.
Non-steroidal anti-inflammatory drugs
*Uric acid-lowering agents*: allopurinol.
*Antiepileptic drugs*: phenytoin, carbamazepine, phenobarbital, lamotrigine, gabapentin, valproic acid.
*Sulfonamides*: dapsone, sulfasalazine, antibacterial and antidiabetic sulfonamides.
*Antivirals*: abacavir, nevirapine, efavirenz.
*Anticoagulants*: heparin, fluindione.
*Cancer immunotherapy*: ipilimumab, nivolumab, pembrolizumab, IL-2, etc.
*Miscellaneous*: dupilumab, synthetic antithyroid drugs, thalidomide, diltiazem, dialysis membranes, iodinated contrast agents, phytotherapy.

Whenever in doubt (and whenever possible), an attempt to discontinue the potential culprit drug/product should be performed. After discontinuation of the causal drug (and initiation of corticosteroids, if appropriate), the clinical course is usually favorable, although it may take some time (up to 6 months) for the CBC to return to normal. Moreover, and relapses may occur upon cessation of local or systemic corticosteroids (if initiated).

If no causal drug is identified, a potential infectious cause for HE (parasitic in particular) should be investigated. As tissue migration induces a Th2-type immune response, the initial stages of helminth infection may be associated with HE and frequently with elevated total IgE levels [48]. Parasite detection is less likely during this phase, and diagnosis relies primarily on serology. HE may then fluctuate or normalize when the adult worms emerge. The etiologies are varied and questioning the patient on his previous travels to parasite-endemic areas. Some parasites can cause chronic infections because of either their long adult life span (e.g., filariasis), self-infestation (e.g., strongyloidiasis) or repeated recontaminations (e.g., pinworm infection). To best guide the etiological investigation for infectious diseases, it is important to ask patients about their dietary habits, hobbies, possible exposure to animals and, of course, history of travel to parasite-endemic areas.

In metropolitan France, the main parasitic infection is toxocariasis, whose clinical presentation ranges from asymptomatic infection to more severe forms [49]. *Toxocara* serology should therefore be routinely performed in all patients with HE > 1.5 × 10^9^/L (symptomatic or not). Nevertheless, differentiating an acute infection from a past infection (“serological scar”) is currently impossible. In addition to *Toxocara* serology, parasitological examination of the stool can allow the diagnosis of pauci-symptomatic or asymptomatic gastrointestinal parasitic infection (mainly ascariasis in metropolitan France) and the confirmation of the diagnosis by direct visualization of the parasite or eggs.

Other investigations should be determined by the risk of parasitic exposure and any clinical and laboratory abnormalities observed. If the patient has spent time in the tropics (even a long time ago), it is advisable to seek the advice of an infectious disease specialist, but strongyloidiasis, filariasis and schistosomiasis should be considered first. The main etiologies of parasitic infection with a cosmopolitan distribution (as well as the modes of infection, characteristics of HE in such situations and corresponding diagnostic methods) are detailed in Additional file 3: Appendix 3.

Noteworthy, other infectious agents that parasites can also cause HE. These include HIV infection (which should be routinely tested for), as well as HTLV-1 infection, for which patients from endemic areas and/or who have spent time in endemic areas (Japan, the Caribbean and sub-Saharan Africa) should be offered a serological test. Finally, note that some protozoan infections acquired in tropical areas (such as malaria, leishmaniasis, amoebiasis and trypanosomiasis) do not cause HE. Once screening for infectious diseases is completed, and in the absence of contraindications, an antiparasitic treatment may be prescribed. The latter can either be:

- Targeted if the parasite has been identified.
- Probabilistic in the presence of a bundle of evidence (clinical, exposure, serology, etc.)
- Empirical, given the possible shortcomings of some parasitological tests.

##### In the event of persistent HE > 1.5 × 10^9^/L with no evidence of either parasitic or drug-induced HE, a diagnostic workup should be performed to guide the choice of treatment.

Any patient with HE > 1.5 × 10^9^/L should undergo an etiological workup including complementary investigations detailed in Box 4. The latter should include a C-reactive protein assay which, if elevated, is suggestive of a solid neoplasm, lymphoma (specifically Hodgkin's and T-cell lymphoma), vasculitis, or certain manifestations of HES (myocarditis, thrombosis). The workup should also include basal tryptase determination (suggestive of mastocytosis or clonal HE/HES), vitamin B12 (elevated B12 and tryptase are suggestive of clonal eosinophilia), plasma protein electrophoresis (polyclonal hypergammaglobulinemia is suggestive of IgG4-related disease, angioimmunoblastic T-cell lymphoma or chronic parasitic infection) and total IgE (although the specificity is limited, elevated levels suggest reactive eosinophilia). A computed tomography (CT) scan of the chest, abdomen and pelvis is also recommended for any patient with persistent HE > 1.5 × 10^9^/L, both to search for a paraneoplastic cause of HE (solid tumor, lymph nodes, as well as splenomegaly in myeloid neoplasms) and to assess the impact of HE (see “Specific settings” section). Note that T-cell phenotyping for abnormal populations (especially CD3− CD4 +), testing for T cell clonality and for the *FIP1L1::PDGRA* fusion gene are not first-line investigations.

**Box 4:** Main investigations to be performed in case of persistent unexplained HE > 1.5 × 10^9^/L

CBC
Serum electrolytes, creatinine
Complete liver function tests
LDH, CPK
Serum calcium and phosphorus
Troponin, BNP
Serum protein electrophoresis
Serum tryptase
Vitamin B12
Total IgE
CRP
HIV serology
*Toxocara* serology
Other serology for parasitic infection and HTLV-1 depending on the context
Parasitological examination of the stool (Baermann method if strongyloidiasis is suspected)
CT of the chest, abdomen, and pelvis

In the absence of a cause and in case of persistent HE, ECG and transthoracic echocardiography should also be performed.

#### Other tests to be performed in the presence of HE > 1.5 × 10^9^/L and a suggestive context

Other investigations should be guided by the context.

##### In case of airways involvement

Mandatory consultation with a pulmonologist.

In case of asthma with typical symptoms (dyspnea, tightness of the chest, cough, variable paroxysmal wheezing, steroid-responsive exacerbations) associated with reversible and variable airway obstruction with HE, the differential diagnoses to be considered are:

- Eosinophilic granulomatosis with polyangiitis (formerly Churg-Strauss syndrome), which should prompt a test for anti-neutrophil cytoplasmic antibodies (ANCA) (present in 30–40% of patients) [50].
- Allergic bronchopulmonary aspergillosis (especially if total IgE > 500 IU/mL and/or proximal bronchiectasis on chest CT), for which *Aspergillus*-specific IgG and IgE assays should be performed [51].
- Eosinophilic asthma alone or associated with chronic rhinosinusitis (with or without polyposis) and without systemic manifestations remains significantly more common than the previous two conditions.
- Eosinophilic bronchitis, bronchiectasis, or bronchiolitis, alone or associated with eosinophilic granulomatosis with polyangiitis or allergic bronchopulmonary aspergillosis.

Pulmonary function tests should be performed as a matter of routine to confirm the diagnosis of asthma (if not previously established) and to assess the severity of airway disorder.

##### In case of parenchymal lung disease

Patients with chronic eosinophilic pneumonia usually present with a chronic cough + / − associated with expectoration and persistent dyspnea (initially exertional and then also at rest). A chest X-ray may show alveolar opacities, most often bilateral, but this examination is not very sensitive.

Thin-section CT scan shows ground-glass and multifocal alveolar opacities, with a usually random and less commonly peripheral distribution, as seen in idiopathic chronic eosinophilic pneumonia (formerly Carrington’s disease) [15]. Exceptionally, nodules may be seen. The chest CT scan may be abnormal even in the absence of respiratory symptoms and should therefore be performed routinely (ideally before initiating corticosteroid therapy).

Pulmonary eosinophilia must be confirmed, usually by bronchoalveolar lavage (BAL) performed during bronchial fibroscopy, showing eosinophilic alveolitis with more than 25% eosinophils and, exceptionally by surgical lung biopsy, whose indication must be discussed in a multidisciplinary team meeting of rare pulmonary diseases. The impact on respiratory function must be assessed with pulmonary function tests (including, spirometry and plethysmography to check for a restrictive ventilatory disorder, diffusion capacity of the lungs for carbon monoxide, and arterial blood gases).

The possibility of drug-induced (iatrogenic) eosinophilic pneumonia, tropical pulmonary eosinophilia (hypersensitivity to *Filariae*) and IgG4-related disease (particularly in the presence of peribronchovascular thickening, mediastinal lymphadenopathy and/or polyclonal hypergammaglobulinemia) should be considered.

##### In case of skin manifestations

Skin biopsy is often essential. In addition, depending on the patient's history and symptoms, a blood smear (seeking for Sézary cells), lymphocyte phenotyping and a blood and cutaneous T-cell clonality assays may also be performed to investigate the presence of cutaneous T cell lymphoma, as well as that of anti–basal membrane antibodies by indirect (blood) or direct immunofluorescence (skin biopsy) if incipient bullous pemphigoid is suspected [52]. In case of fixed urticaria pigmentosa with Darier's sign (suggestive of cutaneous mastocytosis), *KIT* gene mutation may also be searched (both on bone marrow aspiration and skin biopsy), and a tryptase assay may be performed. Eosinophilic fasciitis (Shulman’s syndrome) should be considered in case of thickening of the skin with edema, especially of the forearms, and the diagnosis should be confirmed by magnetic resonance imaging (MRI) of the affected limb, possibly followed by a fascia biopsy [53]. The presence of episodic angioedema with eosinophilia is suggestive of lymphocytic HES (but also of loiasis and onchocerciasis if the patient has a history of travel to a high-risk area) [54, 55]. Finally, nodular lesions due to lymphomatoid papulomatosis are, in the context of blood HE, suggestive of *FIP1L1::PDGFRA*-related HES.

##### In case of inflammatory joint manifestations

In case of polyarthritis and/or tenosynovitis, the presence of ANCA (microscopic polyangiitis, granulomatosis with or without eosinophilic polyangiitis) and anti-cyclic citrullinated peptide antibodies should be investigated (rheumatoid arthritis may in some cases be associated with HE) [56], and blood lymphocyte phenotyping should be performed to search for lymphocytic HES (which is commonly associated with joint symptoms, the causal T-cell population having already been identified in the synovial fluid of patients) [11].

##### In case of lymph node involvement

Seeking for high-grade lymphoma (Hodgkin's lymphoma, angioimmunoblastic T-cell lymphoma or another peripheral T-cell lymphoma) should be performed. In contrast, a more chronic presentation and small lymphadenopathy may suggest lymphocytic HES (the diagnosis of which can be confirmed by peripheral T-cell phenotyping), an overlapping form with IgG4-related disease or Kimura’s disease [57]. In these situations, a CT scan of the chest, abdomen and pelvis should be performed. A complementary positron emission tomography (PET) scan may also be performed at a later stage or immediately in case of suspicious lymphadenopathy. These examinations are used to guide lymph node biopsy, which provides a definitive diagnosis.

##### In case of gastrointestinal symptoms

In addition to parasitic infections and drug-induced eosinophilia, chronic inflammatory bowel disease [58], celiac disease and systemic mastocytosis should be considered (in the latter case, it is worth considering staining with CD117, CD2 and CD25 on gastrointestinal tissue biopsies).

##### In case of arterial ischemic lesions

Depending on the context, a vascular imaging assessment may be performed and/or a dilated fundus examination if cholesterol embolization syndrome is suspected. Note, however, that thromboangiitis obliterans-like lesions and even genuine eosinophilic vasculitis have both been reported in HES [59, 60].

##### In case of rhabdomyolysis and/or myalgia

Laboratory tests for the diagnosis of parasitic diseases should include (in patients with a history of travel to endemic areas and/or exposure) serological testing for trichinellosis and cysticercosis and muscle MRI. Muscle biopsy (mainly to seek for muscle vasculitis) should be considered on a case-by-case basis. Lastly, note that mutations in the *CAPN3* gene (which encodes calpain) causes limb-girdle muscular dystrophy associated with muscle eosinophilia and sometimes moderate blood HE (usually < 1.5 × 10^9^/L) [61].

##### In case of clinical and laboratory signs of inflammation

The search for an underlying solid or hematologic malignancy (especially Hodgkin's or high-grade peripheral T-cell lymphoma) or vasculitis (including testing for ANCA) should be thorough. Indeed, the main types of HES (particularly F/P + chronic eosinophilic leukemia and lymphocytic HES) are not associated with laboratory evidence of inflammation (except for cases of venous or arterial thrombosis, including eosinophilic myocarditis) [62].

##### Features specific to pediatric HE

A consultation with a qualified pediatrician is recommended. In children, HE can be encountered in atopic diseases and tends to parallel with clinical symptoms. Malignant hemopathy should be ruled out by checking for splenomegaly, lymphadenopathy and /or other blood cell count abnormalities (cytopenia, macrocytosis, etc.). A family tree is also essential. Specifically, the diagnosis of hyper IgE syndrome (due to either autosomal dominant *STAT3*, autosomal recessive *DOCK8* or autosomal dominant *IL6ST* mutations, and usually with high IgE levels > 1000 IU/mL) should be considered in patients with a history of neonatal rash, recurrent skin, and pulmonary infections (*Staphylococcus* or *Candida* spp), a suggestive morphotype (facial dysmorphism, high-arched palate), pneumatoceles or retention of primary teeth) [63–65]. Overall, either gastrointestinal involvement and/or growth impairment should raise the possibility of a primary immune deficiency, and serum protein electrophoresis for the determination of IgG, IgA, IgM and IgE should be performed.

### Definition of the type of HE/HES

#### Clonal HE/HES

##### When should it be considered?

Some blood disorders can be associated with HE (either symptomatic or not). Broadly speaking, in addition to certain acute leukemias (particularly FAB M4 acute myeloid leukemia and B-cell acute lymphoblastic leukemia with t(5;14)(q31;q32); *IGH*::*IL3*) [66], there are three conditions that may be responsible for clonal HE:

- Myeloid/lymphoid neoplasms with rearrangements of genes encoding receptor tyrosine kinases (particularly *PDGFRA*, *PDGFRB*, *FGFR1* and *PCM1*::*JAK2*) are constantly associated with HE.
- Some chronic myeloid neoplasms (chronic myeloid leukemia, polycythemia vera, essential thrombocythemia) can also (but are not constantly associated with) lead to HE.
- Finally, chronic eosinophilic leukemia–not otherwise specified (CEL-NOS) is a diagnosis of exclusion made in the absence of any other defined blood neoplasm that may be associated with eosinophilia (including the conditions discussed above, but also systemic mastocytosis and myelodysplastic syndrome). This diagnosis may be made in patients with HES and a high blast count (> 2% in blood or > 5% in bone marrow), or cytogenetic or molecular abnormalities suggestive of a clonal eosinophilia.

Overall, some clinical (hepatomegaly, splenomegaly) and/or laboratory (cytopenia, thrombocytosis, polycythemia, monocytosis, basophilia, elevated serum vitamin B12 and/or tryptase) characteristics and/or the lack of normalization of absolute eosinophil count under treatment with systemic corticosteroids are suggestive of clonal HE. In addition, some clinical manifestations (endomyocardial fibrosis, bilateral border zone strokes, lymphomatoid papulosis, and mucosal ulcerations) are highly suggestive of F/P + chronic eosinophilic leukemia, the latter having a very clear male predominance (M/F sex ratio: 18/1) [19, 39, 67]. Hence, the presence of one or more of these elements should prompt an exhaustive (but sequential, in the absence of therapeutic urgency) seeking for a clonal origin of HE.

##### What first-line investigations should be performed in the presence of clinical and/or laboratory signs suggestive of clonal HE?

- The first test to perform is the search for *FIP1L1::PDGFRA* (F/P) by nested reverse transcription polymerase chain reaction (RT-PCR) or real-time quantitative polymerase chain reaction (RQ-PCR) in peripheral blood, which are more sensitive than fluorescence in situ hybridization (FISH) test to seek for *CHIC2* 4q12 deletion [68]*.* In very rare situations (atypical translocation), the PCR may be falsely negative, in which case FISH may be useful.
- In case of polycythemia and/or thrombocytosis associated with HE, search for *JAK2* V617F, *CALR* and *MPL* mutations in peripheral blood.
- In case of neutrophilia or basophilia associated with HE, test for *BCR::ABL1* in peripheral blood.

##### What tests should be performed if first-line investigations are inconclusive?

If the molecular workup on peripheral blood is negative, a bone marrow aspiration with conventional karyotyping and FISH (to detect translocations involving *ABL1*, *PDGFRB*, *PDGFRA* or *FGFR1*) is recommended. Indeed, these tests can detect chromosomal rearrangements other than *FIP1L1::PDGFRA*, including those involving: (i) platelet-derived growth factor receptor-beta (*PDGFRB*) (including the *ETV6*::*PDGFRB* rearrangement with chromosomal translocation t(5;12)(q32;p13) that can be evidenced on routine karyotyping; (ii) *JAK2*, including the *PCM1::JAK2* rearrangement associated with t(8;9)(p22;p24); (iii) *FLT3*, including the *ETV6::FLT3* rearrangement with t(12;13)(p13;q12); and (iv) *FGFR1* (8p11 myeloproliferative syndrome). The main cytogenetic abnormalities that can cause clonal HE/HES are listed in Additional file 4: Appendix 4. Cytogenetic analysis can also detect non-specific abnormalities (+ 8, del(20q), complex karyotype, etc.) providing a diagnostic component of CEL-NOS [7, 8]. Nevertheless, it should be underlined that the prognostic impact of next-generation sequencing (NGS) abnormalities has not yet been established in HES.

In addition, in case of elevated serum tryptase and negative F/P transcript, it is recommended to test for the *KIT* D816V mutation in bone marrow and to search for abnormal mast cells (CD117+ , CD2+ and/or CD25+) in bone marrow samples. Finally, as detailed in “Pregnancy” Section, it is important to note that laboratory and/or histological markers of mast cell activation (such as the sometimes-dramatic increase in serum tryptase or the presence of dysplastic (spindle-shaped) mast cells on bone marrow biopsy) may be present in F/P + chronic eosinophilic leukemia [19, 69, 70].

##### How useful are NGS-based gene panel tests for investigating HE?

NGS-based gene panel tests may be considered when there is a strong suspicion that HE is clonal, keeping in mind the limitations detailed below. Recent studies have reported the presence of mutations described in myeloid neoplasms (*ASXL1*, *TET2*, *SETBP1*, *CSFR3* and *SF3B1*) in a significant proportion (up to 30%) of patients monitored for unexplained HE [71–73]. In some studies, the presence of a molecular abnormality in these patients was associated with a poorer prognosis [72, 73]. Yet, other studies found no difference in survival between patients with NGS abnormalities compared with those in whom NGS showed no mutations [71].

These data illustrate the obvious methodological limitations of NGS-based approaches in HES:

(i) The presence of these mutations (especially with a low variant allele frequency) is not a surrogate of clonal eosinophilic disease. Indeed, they can also occur with age in the general population (clonal hematopoiesis of undetermined significance, age-related clonal hematopoiesis).
(ii) The clonal nature of eosinophilia is not established, as eosinophilia may be secondary to the production of eosinophilopoietins, such as IL-5, by one or more clonal cells other than eosinophils.
(iii) The therapeutic and prognostic impact of the identification of these mutations in otherwise idiopathic HES patients remains to be determined.

**Fig. 2:**
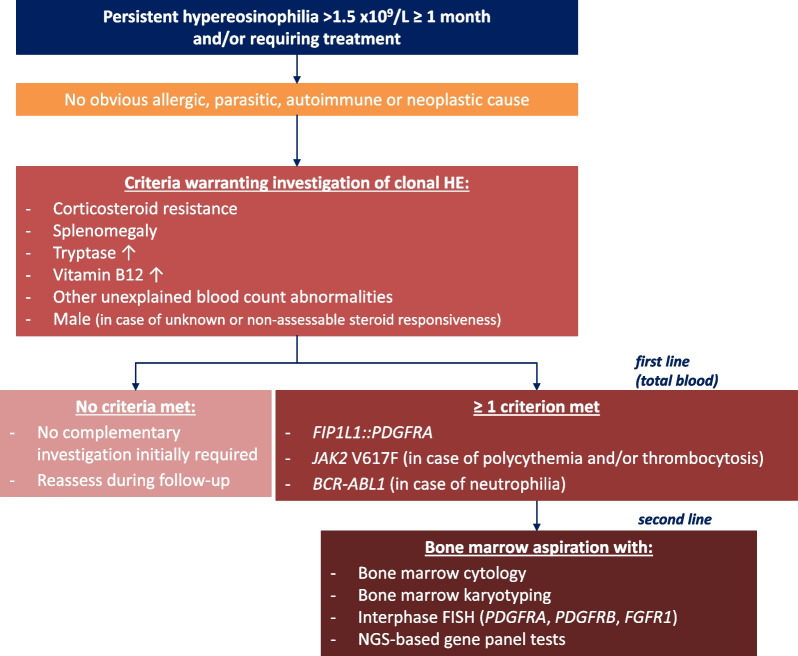
Summary of tests for the investigation of clonal eosinophilia. Abbreviations: FISH, fluorescence in situ hybridization; NGS, next-generation sequencing; PCR, polymerase chain reaction

To avoid the need to perform an additional blood marrow aspiration at a later stage during follow-up in refractory cases, we recommend that bone marrow DNA can be preserved and NGS of bone marrow samples performed at a later stage using the algorithm detailed in Fig. 2.

##### Non-routine tests

- In the absence of clinical or laboratory findings suggestive of F/P + chronic eosinophilic leukemia, the diagnostic yield of F/P testing is low (less than 2% positive results according to a survey of nearly 6700 requests performed in three French university hospitals, unpublished data). The optimization of the prescription of *FIP1L1::PDGFRA* fusion gene is therefore mandatory, and this test should not be included in the first-line workup of cases of all cases of unexplained HE, but rather restricted to the patients who exhibit features suggestive of the disease. In a series of 44 patients with F/P + chronic eosinophilic leukemia for whom the following clinical and laboratory parameters were available (male sex, splenomegaly, tryptase or vitamin B12 elevation), the percentages of patients with 0, 1, 2, 3 or 4 of these parameters were 0%, 2%, 11%, 50% and 36%, respectively. Likewise, in that same series of patients with F/P + chronic eosinophilic leukemia, none of the 31 patients with first-line treatment with systemic corticosteroids normalized their absolute eosinophil counts [19]. Hence, testing for F/P seems irrelevant when the absolute eosinophil count has normalized spontaneously or under corticosteroids alone.
- The indications for diagnostic bone marrow biopsy in HE are limited to patients with suspected aggressive T-cell lymphoma (when other histological biopsies are inconclusive, especially lymph nodes), systemic mastocytosis (although in the latter case, bone marrow mast cell phenotyping and *KIT* mutation testing may be sufficient for diagnostic purposes), or clinical/laboratory findings suggestive of chronic myeloid neoplasm or myelodysplastic syndrome, (when blood marrow aspiration alone is inconclusive). Unlike some other myeloid neoplasms, myelofibrosis identified on bone marrow biopsy has no prognostic impact in patients with F/P + chronic eosinophilic leukemia.

#### Lymphocytic HE/HES

##### When should it be considered?

Lymphocytic HES should be considered in all cases of unexplained or symptomatic HE. Nonetheless, lymphocytic HES is characterized by the high rates of skin (> 80% of patients), lymph nodes (60%), joints (30%, typically bilateral symmetrical nondestructive arthritis, sometimes with associated tenosynovitis), and more rarely gastrointestinal or pulmonary involvements. In contrast, cardiac involvement is exceptional [10, 11].

##### What tests should be performed?

The diagnosis of lymphocytic HE/HES relies on the identification of a circulating abnormal lymphocytic population bearing an aberrant cell-surface phenotype, responsible for the excessive secretion of IL-5 and hence for reactive HE/HES [74, 75].

In daily practice, screening for lymphocytic HES relies on flow cytometry. Ideally, the latter should be performed by a cytometrist familiar with the topic. Involved abnormal lymphocyte populations (and their respective pathological thresholds) include:

- CD3− CD4+ lymphocytes: > 0.5% of total lymphocytes.
- CD3+ CD4+ CD7− lymphocytes: > 6–8% of total lymphocytes before age 60 and > 10% after age 60.
- CD3+ CD4− CD8− TCRαβ+ (T double-negative) lymphocytes: > 1.5% of total lymphocytes.

Note that *treatment with systemic corticosteroids does not alter the data resulting from lymphocyte phenotyping*, especially for CD3− CD4+ lymphocytic HES.

Additionally, the confirmation of lymphocytic HES also relies on:

- Complementary cell-surface markers of involved cells, suggestive a TH2-skewed phenotype (CCR4 + CCR6 − on flow cytometry) [76]. The relevance of other specific markers of Th2 cell subpopulations (CXCR3, CRTH2, etc.) remains to be defined.
- The detection of TCR gene clonal rearrangement. However, the sensitivity of such test may be insufficient for populations involving less than 10% of total lymphocytes. Hence, the absence of TCR gene clonal rearrangement detection does not necessarily rule out the diagnosis of lymphocytic HES. Conversely, the detection of TCR gene clonal rearrangement in the blood alone (that is, without evidence of an abnormal lymphocyte phenotype) is not sufficient to retain the diagnosis of lymphocytic HES per se.

##### What other tests can be performed?

- A CT scan of the chest, abdomen and pelvis performed in the context of HE may show polyadenopathy. A baseline PET scan can be recommended, acknowledging the fact that lymphocytic HES-related lymphadenopathy may be hypermetabolic even in the absence of transformation to high-grade T-cell lymphoma.
- Total serum IgE is increased in 50–70% of cases.
- Serum polyclonal elevation of IgM levels, especially in cases of episodic angioedema with eosinophilia (Gleich's syndrome) [54, 55, 77].
- Cryoglobulinemia may be positive (usually type 3, with low levels, and without any clinical manifestation).
- Biopsies of affected organs (skin, gastrointestinal tract, etc.) may show, in addition to the presence of an excess of eosinophils, infiltration of monotypic CD3+ CD4+ lymphocytes (due to immunohistochemical staining of intracytoplasmic CD3), as well as a clonal rearrangement of the TCR, which can be detected if the lymphocytic infiltrate is abundant. The interpretation of histopathological abnormalities should consider the fact that lymphocytic HES is an indolent CD4+ T-cell lymphoproliferative disorder (with potential lymph node and/or extra–lymph node involvements regardless of the disease transformation into high-grade lymphoma). Furthermore, the presence of an abnormal CD3− CD4+ CD7− or CD3+ CD4+ CD7− T-cell population is not pathognomonic of lymphocytic HES as it can be found in epidermotropic T-cell lymphoma (Sézary syndrome, where the lymphocytic infiltrate is epidermal, whereas it is usually dermal in lymphocytic HES) or angioimmunoblastic lymphoma. In the latter case, the detection of CD10 and/or TFH markers (CXCL13, PD1) is suggestive of angioimmunoblastic lymphoma [74]. Finally, HTLV-1–associated adult T-cell leukemia/lymphoma (ATLL) and some types of peripheral T-cell lymphoma–not otherwise specified (PTCL-NOS) may also involve HE with circulating CD3− CD4+ T cells, and as such also constitute potential differential diagnoses.

Overall, the differential diagnosis between lymphocytic HES and high-grade T-cell lymphoma, particularly angioimmunoblastic T-cell lymphoma, can be challenging. When in doubt, the opinion of a pathologist with expertise in the field should be sought. Some discriminating features are listed in Box 5.

**Box 5:** Main discriminating features between lymphocytic HES and angioimmunoblastic T-cell lymphoma

	L-HES	AITL
General signs	Absent	Present (or initially fluctuating in some cases)
Tumor syndrome	Moderate	Prominent
Polyclonal hypergammaglobulinemia	≈ 50%	50–80%
CRP	Normal	Elevated
Laboratory markers of autoimmunity	Absent	Possible
Hypermetabolism on PET-CT	Possible	Present
*Histological findings*		
CD10	Negative	Positive
CXCL13	Negative	Positive
PD1	Negative	Positive

##### Tests of limited or questionable diagnostic value

- Serum IL-5 assays lack specificity (eosinophils can produce it themselves), and standard assay methods can fail even in lymphocytic HES. In addition, serum IL-5 levels usually plummet rapidly under treatment with systemic corticosteroids.
- Although serum levels of TARC/CCL17 are high in lymphocytic HES (and seem to have a good negative predictive value when levels are < 3000 pg/mL) [75, 78], elevated levels of TARC/CCL17 are not specific enough for the differential diagnosis of HES (e.g., for other lymphoid neoplasms). Moreover, the assay is poorly available in daily practice.

#### Other types of reactive HE/HES

Besides the lymphocytic HE/HES, other conditions (e.g., infectious, dysimmune, neoplastic) can induce the overproduction of eosinophilopoietins (including IL-5) and thus lead to polyclonal reactive HE. When the clinical manifestations are genuinely due to eosinophil toxicity (rather than the underlying disease), the condition is known as reactive HES. Among the causes of reactive HE/HES, recent onset of HE/HES should primarily suggest helminthiasis or drug hypersensitivity, as detailed in “Management of *FIP1L1::PDGFRA*–positive chronic eosinophilic leukemia” section. In case of deterioration of the general condition, laboratory signs of inflammation (which are unusual in HES except in cases of vascular thrombosis including eosinophilic myocarditis), the initial physical examination and whole-body CT scans should strive to seek for findings suggestive of solid (especially (adenocarcinoma) or hematologic (*e.g.,* Hodgkin's lymphoma or peripheral T-cell lymphoma) malignancies.

#### Overlapping conditions

##### Single-organ HES/organ-specific eosinophilic disorders

Current classifications distinguish (yet do not oppose) systemic HES and "organ-specific" eosinophilic disorders (listed in Box 6).

**Box 6:** (Non-exhaustive) list of organ-specific eosinophilic disorders (adapted from Valent et al, JACI [1])

Eosinophilic gastrointestinal disorders
Eosinophilic esophagitis
Eosinophilic gastritis
Eosinophilic enteritis
Eosinophilic colitis
Eosinophilic hepatitis
Eosinophilic cholangitis
Eosinophilic pancreatitis
Eosinophilic ascites
Eosinophilic lung diseases
Hypereosinophilic asthma
Eosinophilic bronchitis and bronchiolitis
Eosinophilic pneumonia
Eosinophilic pleurisy
Eosinophilic interstitial nephritis
Eosinophilic cystitis
Eosinophilic myocarditis
Eosinophilic cellulitis (Wells’ syndrome)
Eosinophilic fasciitis (Shulman’s syndrome)
Eosinophilic folliculitis (Ofuji’s disease)
Eosinophilic synovitis
Eosinophilic vasculitis (localized or systemic)
Other: eosinophilic otitis media, eosinophilic mastitis, eosinophilic endometritis (and myometritis), etc.

Hence, there are several reasons why distinguishing between these conditions is controversial:

- The initial workup for an apparently organ-specific eosinophilic disorder (e.g., chronic eosinophilic pneumonia) should seek for evidence of other potential eosinophil-related organ toxicity (e.g., skin, heart, etc.).
- Such involvement of other organs may only occur at a later stage during follow-up. Hence, only prolonged follow-up can accurately distinguish between an organ-specific eosinophilic disorder and systemic HES with an initial "single-organ" presentation [79].
- Patients with persistent and/or relapsing disease should be monitored carefully in order not to delay the diagnosis of other potential eosinophil-related organ involvement.

Besides very specific cases (e.g., eosinophilic esophagitis in young subjects, usually with no or only mild blood HE; acute eosinophilic pneumonia, eosinophilic cystitis, or "isolated" eosinophilic asthma), we recommend that patients with presumed "organ-specific" eosinophilic disease and HE > 1.5 × 10^9^/L have the same initial workup (both for diagnostic means and assessment of organ involvement, as described in “Pharmacological treatments” Section) as those with systemic multi-organ involvement.

##### Eosinophilic granulomatosis with polyangiitis (formerly Churg-Strauss syndrome)

Although eosinophilic granulomatosis with polyangiitis (EGPA) is classified as an ANCA vasculitis, ANCAs (usually targeting myeloperoxidase) are detected in only about 30% of patients, and their presence tends to correlate with the clinical picture:

- Patients with ANCA have a "vasculitic" phenotype with more frequent alveolar hemorrhage, glomerulonephritis, mononeuritis multiplex and relapses [80, 81].
- Patients without ANCA have an “eosinophilic” phenotype analogous to that of HES (including eosinophilic heart disease, which may affect long-term prognosis) [82].

In addition, recent basic data show that:

- Genetic susceptibility varies depending on the ANCA status (*HLA DQ* is associated with ANCA vasculitis and microscopic polyangiitis, whereas other genes, particularly those associated with the IL-5 pathway, are associated with ANCA-negative forms; finally, other genes, such as *TSLP*, are associated with both ANCA and non-ANCA EGPA) [83].
- The pathophysiology of peripheral neurological damage differs based on the ANCA status, with fibrinoid necrosis being more common in patients with ANCA (whose clinical presentation is most often mononeuritis multiplex, as in other types of small-vessel vasculitis), whereas intraluminal thrombi of the epineural vessels (suggestive of eosinophil-driven toxicity) are more prevalent in ANCA-negative (whose clinical presentation is more likely to be that of sensorimotor polyneuropathy) [84].

It is important to remember that asthma does not make it possible to formally distinguish between EGPA and HES:

- The combination of asthma and chronic rhinosinusitis (with or without nasal polyposis) is common and likely a single disease ("unified airway disease") associated with chronic inflammation of both the upper and lower respiratory tract.
- The available data suggest that approximately a quarter of patients with idiopathic HES may have asthma, including newly diagnosed asthma [85].
- In patients with hypereosinophilic asthma and systemic manifestations, low CRP levels (for reference purposes, < 40 mg/L) suggest HES rather than EGPA [62].

Clinical case definition issues are also complexified by the fact that:

- Histological confirmation of vasculitis is not consistently obtained in reported series of patients with EGPA [80, 81].
- Conversely, eosinophilic vasculitis (localized or systemic) may occur during HES [60].

In daily practice, it can be difficult to differentiate between ANCA-negative EGPA and idiopathic HES (Box 7). Some authors suggested that the term "EGPA" be restricted to patients with either ANCA, histological proof of vasculitis and/or strong clinical surrogates (*e.g.,* extra-capillary glomerulonephritis, intra-alveolar hemorrhage, mononeuritis multiplex, scleritis, purpura) [81], but inclusion criteria for ongoing clinical trials and registries remain flexible [86]. In practice, these clinical case definition issues are likely to ease with the advent of anti-IL5 therapies, which can now be prescribed (rather than conventional immunosuppressants) to both patients with corticosteroid-dependent hypereosinophilic asthma and systemic manifestations, regardless of the diagnostic framework (HES vs. EGPA).

**Box 7:** Key differentiating features between HES and EGPA in patients with hypereosinophilic asthma and systemic manifestations

	ANCA-positive EGPA	ANCA-negative EGPA	HES
ANCA	Positive (almost exclusively MPO)	Absent	Absent
Asthma	Present	Present	Possible
Cardiac involvement	Rare	Common	Possible
Extra-capillary glomerulonephritis	Possible	Absent	Absent
Alveolar hemorrhage	Possible	Absent	Absent
Peripheral neurological presentation	Predominantly single or multiple mononeuropathy	Predominantly polyneuropathy	Predominantly polyneuropathy
Scleritis	Possible	Absent	Absent
Vasculitis	Present	Present	Possible
CRP	Elevated	Variable	Low *(except for thrombosis and severe eosinophilic myocarditis)*

##### IgG4-related disease

Ten to Thirty percentage of cases of IgG4-related disease are associated with HE, possibly the consequence of the Th2 polarization of the immune response reported in this condition. In this setting, HE usually remains moderate, and eosinophil count > 3.0 × 10^9^/L is considered an exclusion criterion according to the latest ACR/EULAR classification criteria [87]. However, it is important to emphasize that these classification criteria were established for epidemiological purposes, as well as to standardize enrollment in clinical trials, but are not intended for routine diagnostic purposes. In addition, some patients with IgG4-related disease can present with massive HE (> 3.0 × 10^9^/L), which may sometimes be symptomatic (cases of eosinophilic cellulitis, thrombosis and eosinophilic pneumonia documented by BAL have been reported) and refractory to treatment targeting the IL-5 pathway, hence with potential diagnostic delay [57]. The diagnosis of IgG4-related disease should therefore be considered in patients with HE in either the presence of either clinical or imaging findings (unexplained pancreatitis, cholangitis, sialadenitis, retroperitoneal fibrosis, etc.) suggestive of IgG4-related disease and/or polyclonal hypergammaglobulinemia.

##### Systemic mastocytosis

Mast cells and eosinophils can interact strongly with one another. Specifically, mast cells can secrete IL-5, prostaglandin D2, VEGF and PAF, which can activate eosinophils. Conversely, eosinophil cationic proteins (such as major basic protein (MBP) and eosinophil cationic protein (ECP)) and stem cell factor (SFC) production by eosinophils may also modulate mast cell activity [88, 89].

F/P + chronic eosinophilic leukemia is commonly associated with the presence of dysplastic (spindle-shaped) mast cells and moderately elevated tryptase (typically < 50 ng/mL) [19, 69, 70].

In addition, the presence of excessive mast cells in tissue biopsies is not uncommon in other types of HES, and if there is any doubt regarding the diagnosis of indolent systemic mastocytosis, specific immunostaining (CD2, CD25) for activated mast cells may be considered.

However, the presence of eosinophilia (or even HE) is possible in genuine systemic mastocytosis and seems to be a negative risk factor [90].

#### Familial HE/HES

Exceptional familial forms of HES and/or EGPA/HES overlap have been reported, with autosomal dominant transmission. Evidence suggests dysregulation of IL-5 production in these steroid-responsive but rarely symptomatic forms [13]. Besides, an autosomal recessive gain-of-function mutation of *JAK1* has also been reported in a mother and her two sons who all presented with prominent HE, atopic dermatitis, and eosinophilic gastrointestinal involvement [30].

#### Idiopathic HES

After ruling out parasitic, drug-induced, inflammatory, and paraneoplastic causes as well as clonal and lymphocytic HES, around 3/4 of cases of HE/HES remain unexplained: these are referred to as idiopathic HES. This diagnosis of exclusion only requires very seldom bone marrow cytology, cytogenetic or molecular testing and the latter should be performed mainly in cases of suspected clonal HE or lymphoma.

#### HE of undetermined significance

HE of undetermined significance (HE_US_) is defined as a condition in which HE is asymptomatic and idiopathic in nature [1, 2, 12]. There is no consensus definition in the literature, but experience in the field suggests that this diagnosis is based on the workup detailed in Box 4 (including lymphocyte phenotyping to search for cell populations of interest in the case of lymphocytic HE/HES, and *FIP1L1::PDGFRA* testing only in the presence of suggestive clinical or laboratory findings). On the other hand, in the absence of clinical or laboratory findings suggestive of clonal HE, bone marrow aspiration and/or biopsy are not mandatory to retain diagnosis of HE_US_.

### Screening for complications of HES

#### General principles

Overall, while there indeed are some preferential associations (e.g., cardiac involvement and clonal HES, angioedema and lymphocytic HES), all clinical manifestations of HES may be seen regardless of the pathophysiological mechanism underlying HE.

Disease staging consists mainly of history-taking, a physical examination and simple, targeted first-line tests. Given the fact that it can be asymptomatic in the initial phase and that the prognosis can be poor when the diagnosis is delayed (potential cardioembolic complications or development of endomyocardial fibrosis), cardiac involvement should be routinely and promptly screened for (even in children). Whenever in doubt, and regardless of the organ involved, appropriate tests should be performed to confirm or rule out the clinical suspicion of eosinophil-related organ involvement. Alternatively, the absence of damage to each system can be checked using the checklist detailed in Additional file 5: Appendix 5.

#### Specificities based on the various types of HE or the patient’s condition and risk factors

##### F/P + chronic eosinophilic leukemia

Given the frequency of microvascular damage related to eosinophil toxicity, it seems appropriate to recommend cardiac and cerebral MRI as first-line examinations (even in asymptomatic patients, and/or if first-line tests, such as troponin/BNP and TTE, are normal).

##### Lymphocytic HES

Given the risk of transformation of lymphocytic HES into high-grade peripheral T-cell lymphoma (particularly angioimmunoblastic T-cell lymphoma), a baseline PET scan should be performed for the diagnosis of L-HES if lymphadenopathy is detected in a CT scan. If there is any uncertainty about whether the disease has progressed to a high-grade hematologic malignancy (see Box 5), a PET/CT-guided lymph node biopsy is also recommended.

### Importance of histological confirmation

Since eosinophilic tissue infiltration is an integral part of the definition of HES, histological confirmation is always desirable. Thus, the purpose of performing a biopsy of potentially affected organ(s) is twofold: first, to support the positive diagnosis of HES, and second, to rule out differential diagnoses. Currently, there are no standardized histological criteria for the definition of pathological eosinophil density in each target organ. The predominance (or abnormally high density, as determined by the pathologist) are usually considered.

## Therapeutic management

### Aims

#### General goals

The goal of the management of HES is to achieve remission of the clinical symptoms associated with HES. In case of severe organ involvement (cardiac or central neurological involvement, thrombosis, etc.), a further goal is to achieve hematologic remission (eosinophil count < 0.5 × 10^9^/L) to minimize the risk of relapse and, above all, of irreversible sequelae [3, 20]. Otherwise (non-serious dermatological, gastrointestinal, or other manifestations), normalization of the eosinophil count is not mandatory in all cases (the main objective rather being in the latter case to control symptoms and monitor the possible occurrence of other clinical manifestations related to eosinophil toxicity).

#### Other specific objectives based on the type of HES

##### Clonal HES

The goal of treatment for clonal HES is to achieve sustained clinical and hematologic (i.e., normalization of the CBC) as well as cytogenetic and/or molecular remission (depending on the underlying disorder identified) in order both to prevent eosinophil-induced organ involvement and transformation into acute leukemia [8, 91].

##### Lymphocytic HES

Although systemic corticosteroids, immunomodulatory or immunosuppressive agents may all decrease the number of abnormal lymphocytes, there is currently no established strategy to eliminate the relevant lymphocyte population.

### Professionals involved

Therapeutic management is generally multidisciplinary, coordinated by a physician with specific expertise in eosinophilic disorders and undertaken in partnership with a reference center for eosinophil-associated disorders (the list of French referral centers is detailed in Additional file 2: Appendix 2 and is available at www.cereo.fr).

It is provided by the same professionals as those involved in the baseline assessment as well as (if needed) other allied health professionals (nutritionists, physiotherapists, psychologists, child psychologists, child psychiatrists) and welfare professionals (social workers, care assistants).

### Pharmacological treatments

#### Antiparasitic treatment

In the absence of available studies, the usefulness of empirical antiparasitic treatment in unexplained chronic HE is disputed. However, we believe that it may be appropriate in specific situations, for the following reasons:

- Variable sensitivity of parasite serology and parasitological examination of the stool.
- Risk of severe strongyloidiasis during treatment with systemic corticosteroids.
- Excellent tolerability of antiparasitic drugs (adverse reactions are exceptional).
- Clinical experience with situations in which empirical antiparasitic treatment allowed complete and sustained normalization of otherwise unexplained HE (despite negative well-conducted parasite tests).
- Low cost.
- When effective, avoids the need for additional, potentially invasive and/or costly second-line investigations.

The initiation of antiparasitic treatment may be contra-indicated or carry a risk of complications in certain situations, including the following: acute schistosomiasis (exposure < 3 months), filariasis, neurocysticercosis or toxocariasis with ophthalmological and/or cardiac involvement. When in doubt, we recommend postponing the initiation of antiparasitic treatment and seeking specialist advice.

##### Empirical antiparasitic treatment in the absence of a history of travel to a specific endemic area

In patients with moderate eosinophilia (0.5–1.5 × 10^9^/L), history-taking must include questions about the shedding of parasites (including in the form of segments: *Taenia*) or itching around the anus mainly at night (pinworm). Empirical antiparasitic treatment with flubendazole (100 mg for 3 days, supplemented by a single dose of 100 mg 15 days later) or albendazole (400 mg/day with meals for 1–3 days, then 400 mg/day at D15) is indicated for any eosinophilia < 1.5 × 10^9^/L in the absence of an obvious cause and contraindications. Main targets: *Oxyuris, Ascaris*.

A single dose of praziquantel (15 mg/kg taken during a meal) is recommended in the presence of eosinophilia with spontaneous passing of segments through the anus or in the stool (to be sent for parasitological examination if possible), even if the parasitological examination of the stool is negative. Main targets: *Taenia*, *Bothriocephalus, Hymenolepis*.

Albendazole (10–15 mg/kg/day, up to a maximum of 800 mg/day, taken twice daily with meals, for 10–15 days) is recommended for unexplained HE > 1.5 × 10^9^/L. Main targets: *Toxocariasis, trichinosis, ascariasis, pinworm infection* (do not prescribe flubendazole in this case).

##### Empirical antiparasitic treatment in case of history of travel to an endemic area (African continent, including the Maghreb and Middle East, Southeast Asia, Central and South America, Caribbean and Indian and Pacific Oceans)

This recommendation applies even to stays that date back more than 20 years, and only after investigation and consultation with a parasitologist. Depending on the patient's condition and risk factors and the endemic areas visited, an antiparasitic treatment consisting of all or some of the following compounds may be offered:

- D1 ivermectin: 200 µg/kg on an empty stomach (possibly followed by a second dose at D2 or D15 in case of diagnostic confirmation of strongyloidiasis).

Main targets: *Strongyloides* (also present in Spain, Portugal, Italy, Eastern Europe, and USA), causative agents of filariasis.

- D2 praziquantel: 40 mg/kg single dose after the evening meal.

Additional targets: Schistosomiasis, certain trematode infections.

- D3–D18 albendazole: 10–15 mg/kg/day, up to a maximum of 800 mg/day, taken twice daily with meals for 5–15 days.

Main targets: *Toxocariasis*, *hookworm infection*, *pinworm infection*, *ascariasis*,* trichuriasis.*

Note: modalities for the treatment of toxocariasis are not standardized (whether in terms of dose or duration), but treatment with albendazole (10–15 mg/kg/day, up to a maximum of 800 mg/day) for 10 days seems to be appropriate. In case of strong suspicion of toxocariasis and failure of first-line treatment with albendazole, second-line treatment with diethylcarbamazine may be considered on a case-by-case basis after consultation with an infectious disease specialist or parasitologist.

#### Indications, modalities, and measures associated with systemic corticosteroid therapy

##### Indications and modalities

Systemic corticosteroid therapy is indicated in the following situations:

- Life-threatening emergency situations.
- First-line treatment for lymphocytic or idiopathic HES.
- As a one-time therapeutic test to assess HE response to corticosteroids.

In an emergency situation (myocarditis, acute respiratory distress, central or peripheral neurological involvement, venous or arterial thrombosis, etc.), regardless of the cause of HES (including parasitic causes, with the exception of severe strongyloidiasis), the initial management of severe visceral involvement secondary to eosinophil toxicity relies primarily on corticosteroid therapy: 1 mg/kg/day prednisone, possibly preceded by intravenous pulses of methylprednisolone (5–15 mg/kg/day, up to a maximum of 1000 mg for 3 days), while awaiting the initial diagnostic workup, which then allows targeted management to be offered if necessary.

Therapeutic test with corticosteroids. For the diagnostic workup of unexplained HE, in the absence of clear characterization of the etiology of HE despite a well-conducted first-line workup (as detailed in Box 4), and in case of persistent HE despite antiparasitic treatment, assessment of the corticosteroid sensitivity of HE (CBC at D3 and D7) following short-term empirical corticosteroid therapy (e.g., 0.5–1 mg/kg/day for 7 days) may be useful (even in the absence of an otherwise indication for long-term treatment with corticosteroids).

The purpose of this therapeutic test is twofold:

- To guide the indication of molecular blood tests: if the eosinophilia is fully steroid-sensitive, tests for clonal HE/HES (which is usually steroid-resistant), may reasonably be waived.
- Confirm that corticosteroids will be effective in the event of a subsequent acute exacerbation of HES requiring systemic therapy.

##### Measures related to corticosteroid therapy

Preemptive treatment with ivermectin (200 µg/kg on an empty stomach) is recommended (in the absence of contraindications) for prophylaxis of severe strongyloidiasis.

If corticosteroid therapy is prolonged, standard practices associated with the prescription of corticosteroid therapy should be applied, including:

- Compliance with health and dietary rules (adequate calcium and vitamin D intake, limitation of overall calorie intake to prevent weight gain, low-carbohydrate diet with high glycemic index to prevent steroid-induced diabetes, low-sodium diet only in case of poorly controlled arterial hypertension and/or heart or kidney failure and/or portal hypertension, physical activity to mitigate steroid myopathy and prevent potential metabolic adverse reactions of steroids).
- Prevention of steroid-induced osteoporosis according to current recommendations (including the prescription of bisphosphonates when indicated) and in the absence of contraindications.
- Prevention of infectious complications on a case-by-case basis (presence or absence of underlying lung disease, underlying immunodeficiency, dose, and duration of corticosteroid therapy, etc.). This can involve ensuring that all vaccinations are up to date, annual influenza vaccination, prime-boost pneumococcal vaccination (13-valent conjugate vaccine followed at least 8 weeks later by a 23-valent polysaccharide vaccine), vaccination against SARS-CoV-2 (whereas live attenuated vaccines are contraindicated in patients receiving corticosteroid therapy at doses ≥ 10 mg/day prednisone-equivalent for ≥ 15 days), prevention of HBV reactivation, prevention of TB reactivation, and possible initiation of pneumocystis pneumonia prophylaxis.
- Screening and management of cardiovascular risk factors.

#### Management of *FIP1L1::PDGFRA–*positive chronic eosinophilic leukemia

##### Indications

Given the risk of both irreversible tissue damage (particularly cardiac) in persistent HE and of transformation into acute leukemia, it is recommended that treatment be initiated in any patient with F/P + chronic eosinophilic leukemia, symptomatic or not [91]. In addition, as the time to initiation of treatment appears to be correlated with the risk of relapse following attempts to discontinue treatment, it is recommended that treatment be initiated promptly once a diagnosis of F/P + chronic eosinophilic leukemia has been made [19].

##### First-line treatment

First-line treatment consists of imatinib, a tyrosine kinase inhibitor, which achieves laboratory and molecular remission in 100% of cases in our experience. A dose of 100 mg daily is sufficient to achieve a complete response, while improving tolerability [19, 92]. The introduction of imatinib may be recommended even before the result of the *FIP1L1::PDGFRA* test when the patient's condition is severe, and the clinical and laboratory findings are suggestive of such diagnosis.

It is important to note that treatment with imatinib requires the use of both male and female contraception. The practicalities of prescribing imatinib are detailed in Box 8.

**Box 8:** Prescribing guidelines for imatinib

Imatinib is administered at a dosage of 100–400 mg/day (to be adjusted in children) depending on the molecular abnormality involved (100 mg/day for* PDGFRA* rearrangement, 400 mg/day for *PDGFRB* rearrangement), taken as a single oral dose with a meal (to improve gastrointestinal tolerability).
In case of specific cardiac involvement related to eosinophilic heart disease, as some cases of paradoxical worsening have been reported at the initial phase of treatment (tumor lysis syndrome), a short course of oral corticosteroids (e.g., 1 mg/kg/day for 5–7 days) may also be prescribed jointly initially. Because of its potential teratogenic effects, contraception (in both female and male) is mandatory for all patients of childbearing potential receiving imatinib, as is adequate photoprotection (risk of photosensitivity).
Imatinib is generally well tolerated, likely since the dosages are lower than those used for the treatment of chronic myeloid leukemia. The main potential adverse reactions are musculoskeletal pain, gastrointestinal disorders, photosensitivity, cytopenia, edema, abnormal liver function tests and growth retardation in children. These adverse reactions should be screened for at each follow-up visit and call for require regular laboratory monitoring (e.g., every 2 weeks for the first 2 months, and then every 3 months thereafter). In case of laboratory abnormalities suggestive of imatinib toxicity (e.g., severe neutropenia < 1.0 × 10^9^/L, thrombocytopenia < 50 × 10^9^/L, cytolysis >5ULN, etc.), temporary cessation of therapy is recommended. Finally, since cases of viral reactivation (sometimes with fulminant hepatitis) have been reported in HBV-positive patients treated with imatinib, HBV viral load monitoring is recommended at the start of treatment and semiannually thereafter in patients with anti-HBc antibodies.
When imatinib is co-administered with other drugs, drug interactions may occur. In particular, imatinib is a substrate for s-glycoprotein and is metabolized by cytochrome 3A4 (CYP3A4); caution should therefore be exercised when imatinib is prescribed in combination with CYP3A4 inducers (including rifampicin, phenobarbital, carbamazepine and phenytoin), CYP3A4 inhibitors (protease inhibitors, azole drugs, macrolides, amiodarone, diltiazem, vancomycin, etc., risk of decreased effectiveness of imatinib), CYP3A4 inhibitors (protease inhibitors, azole drugs, macrolides, amiodarone, diltiazem, verapamil, etc., increased risk of adverse reactions from imatinib), or with other CYP3A4 substrates with a narrow therapeutic margin (e.g., anticalcineurins, sirolimus, warfarin and other coumarin derivatives, and digoxin).

In case of HES-specific cardiac involvement, there is a theoretical risk of initial worsening upon initiation of imatinib (toxicity induced by eosinophil lysis during treatment). This warrants laboratory monitoring of troponin 48 h after initiation of treatment (which should be done in hospital in case of severe eosinophilic heart disease), and the prescription of a short course of corticosteroids if troponin increases during imatinib treatment (e.g., 1 mg/kg/day corticosteroids for 5–7 days).

When F/P + leukemia is diagnosed in the acute phase (acute leukemia) or in the form of other aggressive hematological presentations (lymphoblastic lymphoma), treatment should be discussed in a multidisciplinary team meeting of experts in hematology. Indeed, while molecular remission has been reported in some patients treated with imatinib monotherapy, the addition of a combination of cytarabine and daunorubicin in the initial phase of treatment should be considered depending on the patient's condition [91, 93]. Once molecular remission has been achieved, maintenance therapy with imatinib monotherapy usually makes it possible to defer first-line allogeneic bone marrow transplantation.

##### What to do in case of non-response or secondary loss of response after first-line treatment with imatinib

In case of lack of effectiveness of imatinib, the two most common scenario to consider are:

- Non-compliance to treatment (or a pharmacokinetic issue related to imatinib malabsorption). Although no target residual plasma levels are defined in the context of F/P + chronic eosinophilic leukemia, undetectable drug plasma levels can avoid unnecessary sequencing of *PDGFRA*.
- Another cause of HE (other than F/P + chronic eosinophilic leukemia) that should warrant minimal reinvestigation (as detailed in “Pharmacological treatments” Section).

In addition, there have been exceptional reports in the literature of mutations of the *PDGFRA* gene (particularly the T674I—which is analogous to the T315I mutation in *BCR::ABL1*, and D842V mutations) that confer resistance to imatinib. Second-generation tyrosine kinase inhibitors (TKIs), such as dasatinib and nilotinib, have been shown to be effective *in vitro* on both wild-type and mutated tyrosine kinases, and treatment success has been described with both compounds in this situation [94]. To date, given the rarity of the situation, there is no robust data favoring one or the other compound, and the choice should be based primarily on the tolerability profile and the physician's prescribing habits.

##### Duration of treatment

There have been reports of sustained remission after cessation of imatinib (possibly permanent cure). Our experience (which is consistent with other data in the literature) suggests that ≈ 40% of patients can maintain sustained molecular remission after initial discontinuation of imatinib [19, 95].

In the largest reported case series of 151 patients with F/P + chronic eosinophilic leukemia (46 of whom discontinued treatment with imatinib), the factors that correlated with the risk of relapse following cessation of imatinib were a long delay between the onset of HE and initiation of imatinib, and a short duration of treatment [19]. Treatment with imatinib 100 mg/day can be administered on a long-term basis, but cessation can also be attempted on a case-by-case basis after a minimum of 5 years of treatment in patients in prolonged molecular remission. Regular molecular follow-up (even in the absence of recurrence of eosinophilia) is mandatory (*e.g.*, every 3 months in the first year and every 6 months thereafter). Unlike chronic myeloid leukemia, a consensus definition of molecular remission in F/P + chronic eosinophilic leukemia is lacking. Currently, it relies either on a negative RT-PCR or Q-PCR assay.

In the event of clinical/hematological/molecular relapse after a first attempt to discontinue treatment with imatinib, resumption of treatment usually leads to renewed molecular remission (although rare cases of secondary resistance have been reported). At present, data regarding second attempts to discontinue treatment are scarce.

#### Management of non-F/P-associated clonal HES

The management of non-F/P-associated clonal HES is not standardized, and literature data are even sparser than those available for F/P + chronic eosinophilic leukemia [8, 91]. The therapeutic approach generally depends on the molecular/cytogenetic abnormality reported, and discussion in a multidisciplinary team meeting is recommended. Nonetheless, a few trends emerge.

##### Rearrangements involving *PDGFRA* (other than *FIP1L1::PDGFRA*)

Clonal forms of HES involving *PDGFRA* and a partner gene (such as *BCR, ETV6, KIF5B,* etc.) other than *FIP1L1* are possible. Although exceedingly rare, and by analogy with F/P + chronic eosinophilic leukemia, they are also particularly sensitive to low dose imatinib (i.e., 100 mg daily), and their long-term prognosis seems to be excellent [8, 91].

##### Rearrangements involving *PDGFRB*

Rearrangements involving *PDGFRB* and other partners (about 30 to date) have been reported, including *ETV6::PDGFRB*, which may also be responsible for a chronic myelomonocytic leukemia phenotype with HE due to t(5;12)(q32;p13). These cases are generally responsive to imatinib (MA), but initially at higher doses (400 mg daily) than those used to treat F/P + chronic eosinophilic leukemia [33, 91]. These dosages generally result in prolonged molecular remission with continued treatment. Once molecular remission is achieved, careful reduction of the doses to 100 mg imatinib may be suggested, subject to regular molecular monitoring.

##### *JAK2* rearrangements

*JAK2* rearrangements (including *PCM1::JAK2* due to t(8;9)(p22;p24) and *BCR::JAK2* due to t(9;22)(p24;q11)) are sensitive to JAK2 inhibitors, including ruxolitinib (off label). However, response to treatment may be transient and this therapeutic approach is usually a bridge to allogeneic bone marrow transplantation, which should be considered (depending on the patients’ age and comorbidities) [91, 96].

##### *FGFR1* rearrangements

*FGFR1* rearrangements, including *FGFR1::ZMYM2* due to t(8;13)(p11;q12), are associated with aggressive clinical phenotypes with, in the absence of treatment, constant and rapid progression (in one to two years) towards a high-grade hematological malignancy (acute myeloid leukemia, lymphoblastic lymphoma) resistant to standard TKIs (including ponatinib). In the FIGHT-203 study, a phase 2 multicenter trial assessing the safety and efficacy of pemigatinib (13.5 mg QD on a 21-day cycle with 2 weeks on and 1 week off)), complete hematological and cytological response rates reached 76% and 71%, respectively in 35 previously treated patients in both chronic and blast phase. Additionally, both treatment-naive patients on chronic phase achieved hematological and cytological responses, while one in three treatment-naive patients with blast phase disease achieved hematological and cytological responses. Overall, despite high rates of treatment-emergent adverse events (with hyperphosphatemia, alopecia, diarrhea and stomatitis being at the forefront), pemigatinib may offer a long-term treatment option for patients ineligible for hematopoietic stem cell transplantation, or may otherwise facilitate bridging to transplantation [97]. Of note, pemigatinib was approved by the FDA on August 2022 in this setting.

##### *FLT3* rearrangements

Recently, *FLT3* rearrangements have been included within the subgroup of “Myeloid/lymphoid neoplasms with eosinophilia and tyrosine kinase gene fusions” in the 2022 International Consensus Classification of myeloid neoplasms and acute leukemias [6]. Patients with *FLT3* rearrangements generally have an aggressive phenotype resistant to the main TKI. Thus, as for rearrangements involving *FGFR1*, it seems appropriate to encourage enrollment in clinical trials assessing tyrosine kinase inhibitors showing *in vitro* activity against *FLT3* (sorafenib, sunitinib, gilteritinib) either alone (in case of diagnosis at a chronic phase) or in combination with intensive multiagent chemotherapy, followed by early allogeneic bone marrow transplantation (when feasible) [91].

##### Chronic eosinophilic leukemia–not otherwise specified (CEL-NOS)

Although this is likely a heterogeneous condition (especially when the presumed clonality of eosinophils is based "only" on the presence of cytogenetic (karyotype / FISH) and/or molecular (NGS-based gene panel test) abnormalities, it appears to have a poor prognosis, with high rates of progression to acute myeloid leukemia in the main series reported to date. Thus, a case-by-case assessment considering the patient's cytogenetic and molecular findings is recommended. Case reports (and CEREO experience) have shown that probabilistic treatment with tyrosine kinase inhibitors (imatinib as well as second-generation TKIs or JAK inhibitors) can sometimes elicit a complete hematologic response in this setting, including in patients with no overt molecular abnormality. Empirical (or even sequential, if first-line treatment with imatinib fails) use of these treatments may be considered. In young people, if this strategy fails (and/or if there is cytogenetic evidence of a poor prognosis), allogeneic bone marrow transplantation should also be considered. Cytoreductive therapy with hydroxycarbamide (off-label) is often administered to reduce blood HE (and possibly limit eosinophil toxicity) even though its effectiveness on the natural course of the blood disorder (including the risk of transformation to acute myeloid leukemia) has not been demonstrated. Finally, in the case of multiple documented molecular abnormalities with a significant allele frequency (≥ 5%) and/or a borderline form with a myelodysplastic syndrome, treatment with a hypomethylating agent (azacytidine, off-label) or allogeneic bone marrow transplantation may also be considered on a case-by-case basis [8, 98].

#### Management of idiopathic HES (whether single-organ or systemic)

##### Indications

In case of recurrent transient paroxysmal non-severe manifestations (such as most cutaneous or gastrointestinal symptoms), short courses of corticosteroids (topical or systemic) are usually preferred to long-term exposure to treatment.

Conversely, long-term treatment is required in case of severe and/or disabling clinical manifestations, or in case of recurrent paroxysmal events.

##### First-line treatment: topical and/or systemic corticosteroid therapy

Corticosteroid therapy is generally the first-line treatment for idiopathic HES, and rapid response (complete or partial) is the norm.

Depending on the clinical manifestations, the use of locally active steroids (topical corticosteroids, orodispersible budesonide for esophagitis, gastro-resistant budesonide for lower GI manifestations and inhaled corticosteroid therapy or nasal spray) is encouraged to reduce the use of systemic corticosteroids.

When organs not accessible (or resistant) to topical corticosteroids are involved, systemic corticosteroids are the first-line treatment for idiopathic HES. The loading dose (0.5–1 mg/kg/day prednisone (off-label)), possibly preceded by pulses of methylprednisolone (15 mg/kg/day for 1–3 days) in case of organ- or life-threatening manifestations, should be tailored to the severity of symptoms before gradual tapering. Overall, the minimum effective dose should be used, and weaning should always be discussed: in acute forms, cessation of corticosteroid therapy may be attempted after a few weeks of treatment; in the case of chronic disease, a treatment period of approximately 12 months seems advisable before considering cessation.

##### Second-line treatments

To minimize the potential adverse reactions of systemic corticosteroid therapy, steroid-sparing treatment may be considered in patients with high dose corticosteroid dependence, considering the patient's specific comorbidities).

To date, being the only drug having been assessed in prospective randomized controlled trials, we currently recommend mepolizumab (300 mg subcutaneous monthly) as the first-line steroid-sparing agent. Contrariwise, all other compounds are prescribed off-label based essentially on the available retrospective data.

Following two clinical trials that demonstrated the superiority of mepolizumab, an IgG1 kappa monoclonal antibody targeting IL-5, vs. placebo in terms of reduction of the number of clinical relapses and steroid-sparing effect, mepolizumab was approved by the FDA in September 2020, by the EMA in November 2021 [99, 100]. Conversely, although preliminary data suggest that mepolizumab could remain efficacious even at lower “asthma” doses (i.e. 100 mg subcutaneous monthly) in other eosinophil-associated diseases (*e.g.* EGPA or idiopathic chronic eosinophilic pneumonia), it should be emphasized that, to date, standard treatment of HES relies on a full dose regimen i.e. 300 mg mepolizumab subcutaneous monthly [101].

Specifically, we suggest using mepolizumab in the following settings:

- Chronic AND idiopathic AND oral-corticosteroid-dependent HES (meaning that the efficacy of oral corticosteroids has previously been established, and that an attempt to withdraw oral corticosteroids has been followed by both a clinical AND a hematological relapse)
- Relapsing (either single-organ OR systemic) HES with at least 3 yearly flares requiring short courses of oral costicosteroids.

Under treatment with mepolizumab, once that sustained clinical and hematological remission have been achieved, the tapering (or even withdrawal) of both oral corticosteroids and other cytotoxic-immunosuppressive drugs is strongly encouraged.

Contrariwise, it should also be emphasized that there is to date no rationale for the prescription of mepolizumab either as first-line therapy (even in the setting of organ or life-threatening HES—where systemic corticosteroids, along with antiparasitic treatments and anticoagulants are usually effective, as detailed in “Follow-up of F/P + HE/HES” Section). Likewise, in patients under low dose oral corticosteroids and with no treatment-related side effect, maintenance treatment with low dose oral corticosteroids monotherapy is acceptable.

In case of failure of mepolizumab, further-line steroid-sparing treatments include:

- Interferon alfa-2a, an immunomodulatory agent acting on both eosinophilia and Th2-polarized T cells [102]. Currently, only the pegylated form is available (Pegasys^®^). Although the reported efficacy is relatively high, its safety profile can be a barrier to its use and long-term maintenance [103]. It is therefore standard practice to start with the lowest possible dosage (e.g., 45 µg pegylated interferon alfa-2a weekly by subcutaneous injection) and to increase it gradually monthly if necessary, depending on tolerability. The practicalities of prescribing pegylated interferon alfa-2a are detailed in Box 9.
- Hydroxycarbamide, a non-specific myelotoxic agent with anti-eosinophilic activity. In practice, it is usually started at a low dosage (1 g/day in a single dose) and then increased if necessary (up to 2 g/day in a single dose) depending on effectiveness and tolerability [8]. The practicalities of prescribing hydroxycarbamide are detailed in Box 10.
- Although its primary indication is clonal HES (specifically F/P + chronic eosinophilic leukemia), imatinib (400 mg/day) may also be used empirically (and off label) in patients with laboratory and clinical findings suggestive of underlying myeloid malignancies (although by definition, idiopathic HES does not show the cytogenetic or molecular abnormalities characteristic of clonal HES) *e.g.* splenomegaly, elevated vitamin B12 or tryptase, presence of other CBC abnormalities, nonresponse to corticosteroid therapy (or corticosteroid dependence on more than 10 mg/day prednisone) [104]. In the absence of response at 1 month after initiation of imatinib, the latter should be discontinued and replaced by another steroid-sparing drug.
- Conventional immunosuppressive agents (cyclosporine, methotrexate, and mycophenolate mofetil): limited data are available regarding their benefit in HES. For the time being, their use in this indication is limited.
- Recent preliminary data suggest that benralizumab (a monoclonal antibody targeting the alpha subunit of IL-5 receptor, IL-5Rα) could be a promising treatment for HES [105], and a prospective randomized placebo-controlled trial is underway.

**Box 9:** Prescribing guidelines for pegylated interferon alfa-2a

Pegylated interferon alfa-2a is administered by weekly subcutaneous injections. In HES, common practice is to start with low doses (e.g., 45 μg per week, less than those used for myeloid neoplasms or viral hepatitis) to promote tolerability and long-term adherence. Combination with prophylactic administration of paracetamol (1 g every 8 hours for 24–48 hours) should be offered to reduce flu-like symptoms that can sometimes be observed after injection.
Pegylated interferon alfa-2a is contraindicated in children under 3 years of age, in case of documented hypersensitivity to benzyl alcohol, severe decompensated liver failure, thrombocytopenia < 100 × 10^9^/L or progressive autoimmune disease (particularly systemic lupus erythematosus, dermatomyositis, other diseases mediated by interferon pathways and autoimmune hepatitis). Overall, if there is a history of preexisting autoimmune disease, the benefit/risk ratio of treatment with pegylated interferon alfa-2a should be weighed on a case-by-case basis. In patients with a history of mood and/or personality disorders, a psychiatrist should be consulted prior to initiation of treatment (and monitoring increased if necessary), as this drug may induce mood disorders.
In addition, regular laboratory monitoring (including CBC, serum electrolytes, creatinine, transaminases, TSH) is recommended. In the event of cytopenia, the intervals between injections of pegylated interferon alfa-2a may be increased and/or the dosages reduced.

**Box 10:** Prescribing guidelines for hydroxycarbamide

Hydroxycarbamide is administered orally at a daily dosage of 15–30 mg/kg (in one or two daily doses). Outside of emergency settings (where the starting dose is typically 1500–2000 mg daily), the standard practice for HES is to start with a low dosage (e.g., 1000 mg daily) and then increase it as needed depending on treatment effectiveness and tolerability. Hydroxycarbamide is contraindicated in patients with lactase deficiency, pregnancy and severe cytopenia (neutropenia < 10 × 10^9^/L, thrombocytopenia < 100 × 10^9^/L and/or anemia < 10 g/dL).
Because the risk of bone marrow failure is higher in patients who have received prior radiation or chemotherapy, hydroxycarbamide should be administered with caution in this situation. Likewise, in case of renal failure and/or concomitant use of other myelosuppressive agents, a reduction in the dosages of hydroxycarbamide and increased monitoring are recommended.
In case of significant HE, significant splenomegaly and/or elevated uric acid levels, hydration is recommended at the start of treatment to prevent the possible occurrence of lysis syndrome.
Regular (e.g., every two weeks for two months after initiation of treatment, and then every three months thereafter) clinical (including screening for oral or photo-induced lesions, trophic skin disorders, skin neoplasia and/or fibrosing lung disease that may be promoted by treatment) and laboratory monitoring (including CBC, serum electrolytes, urea, creatinine, transaminases, etc.) is indicated in long-term treatment. Hydroxycarbamide treatment is known to be associated with an increase of erythrocytes’ average globular volume of to up to 120 fL. Lastly, live attenuated vaccines are contraindicated in case of concomitant treatment with hydroxycarbamide.

In daily practice, pegylated interferon alfa-2a is often favored in young patients because of the theoretical long-term leukemogenic risk of hydroxycarbamide, and the need for male and female contraception during treatment and up to 3–6 months after cessation. However, the indications for treatment with pegylated interferon alfa-2a must be assessed on a case-by-case basis, particularly in patients with a significant psychiatric history, liver disease and/or a history of concurrent underlying autoimmune disease (particularly lupus).

#### Therapeutic management of lymphocytic HES

##### Indications

If a close clinical and laboratory monitoring is performed, non-treatment is perfectly acceptable in asymptomatic patients with lymphocytic HE.

In the event of clinical manifestations attributable to eosinophil toxicity and/or to the abnormal T-cell population (lymphocytic HES), treatment strategies (with a preference for topical corticosteroids and short-term rather than long-term treatment) share similarities with those previously described for the management of idiopathic HES, with the noteworthy exception that there is solid data supporting the use of Interferon alfa-2a in this setting.

##### First-line treatments

In general, lymphocytic HES is highly responsive to corticosteroids.

Topical corticosteroids can be effective on localized inflammatory lesions, keeping in mind the cumulative dose.

Inhaled corticosteroids are recommended for recurrent and/or persistent moderate respiratory manifestations.

Oral corticosteroid therapy is the usual first-line treatment of lymphocytic HES. The loading dose (0.5–1 mg/kg/day prednisone) should be tailored to the severity of the symptoms related to eosinophilia, before gradual tapering. Overall, the lowest effective dose should be used and weaning attempted as early as possible.

##### Second-line treatments

Mirroring the fact that lymphocytic HES is usually highly sensitive to systemic corticosteroids, high-dose steroid dependence is also common (possibly due to high levels of serum IL-5 and/or persistent underlying T-cell lymphoproliferative disease). To minimize the potential adverse reactions of systemic corticosteroids, steroid-sparing treatment may be considered in steroid-dependent patients.

Currently mepolizumab and interferon alfa-2a are the preferred first-line steroid-sparing treatments for lymphocytic-HES patients with high-dose corticosteroid dependency.

- Interferon alfa-2a, an immunomodulatory agent acting on both eosinophils and Th2-polarized T cells. Retrospective of both series Both Belgian, Canadian, and French retrospective showed marked clinical and hematological efficacy [10, 11, 106]. Nevertheless, the drug tolerability can be a barrier to its use and long-term maintenance. In daily practice, to minimize potential side effects and to promote adherence to treatment, we suggest starting at the lowest possible dose of the pegylated form (e.g., weekly subcutaneous injections 45 µg pegylated interferon alfa-2a) and to increase it gradually based on tolerability.
- Mepolizumab has also showed efficacy and steroid-sparing effect in lymphocytic HES, with a presumed better safety profile than interferon [107]. Yet persistent symptoms (despite normal absolute eosinophil counts) have also been reported [11].

In case of failure of both mepolizumab and interferon, further-line steroid-sparing drugs may be discussed in expert centers. These include:

- A few cases of successful treatment of refractory lymphocytic HES with romidepsin have been reported [108]. However, the small number of cases (n < 5) and short follow-up do not currently make it possible to clearly define the place of this compound in the therapeutic arsenal against lymphoid variant of HES, the indication of which may be discussed in a multidisciplinary team meeting.
- The membrane expression of CCR4 by clonal T cells suggests that mogamulizumab may be of interest, but its relevance remains to be determined.
- Following the identification of acquired abnormalities in factors affecting the JAK-STAT signaling pathway (including gain-of-function mutations of *STAT3*) in lymphocytic HES [108, 109], the potential effectiveness of JAK inhibitors (ruxolitinib and tofacitinib) has been suggested in small case series, surprisingly even in the absence of gene mutations of the JAK/STAT pathway [110, 111].
- Despite an attractive rationale for their use, limited data are available on immunosuppressive agents that can target T cells, such as cyclosporine, methotrexate, and mycophenolate mofetil.

#### Therapeutic management of reactive HES (other than lymphocytic)

As a general rule, when an underlying factor is identified as the cause of HE/HES, its management is based on the etiological treatment of the underlying disorder (discontinuation of the causal drug/antiparasitic treatment/chemotherapy).

In addition, in the event of organ involvement related to eosinophil toxicity, oral corticosteroid therapy may be recommended at the acute phase, pending the effects of the etiological treatment, and with the aim of weaning the patient from systemic steroids within than three months, if possible.

#### Management of HE of undetermined significance

The management of HE_US_ is not standardized. Some teams have reported prolonged follow-up (several years) in patients with significant HE (sometimes > 5.0 × 10^9^/L in the absence of any treatment [12]. However, the time between the onset of HE and the first related clinical manifestation can also be prolonged. The therapeutic approach should therefore be discussed with patients on a case-by-case basis, depending on their possible comorbidities, their understanding of the situation and their compliance with follow-up. Although there is no strict correlation between the levels of blood HE and tissue HE, it is conceivable not to initiate treatment in patients with asymptomatic HE < 5.0 × 10^9^/L but to continue long-term monitoring as described in “Follow-up” section. In asymptomatic clonal (or presumed clonal) HE, a multidisciplinary team meeting on whether treatment is indicated should be considered.

### Specific settings

#### Organ or life-threatening HES involvement

**Fig. 3:**
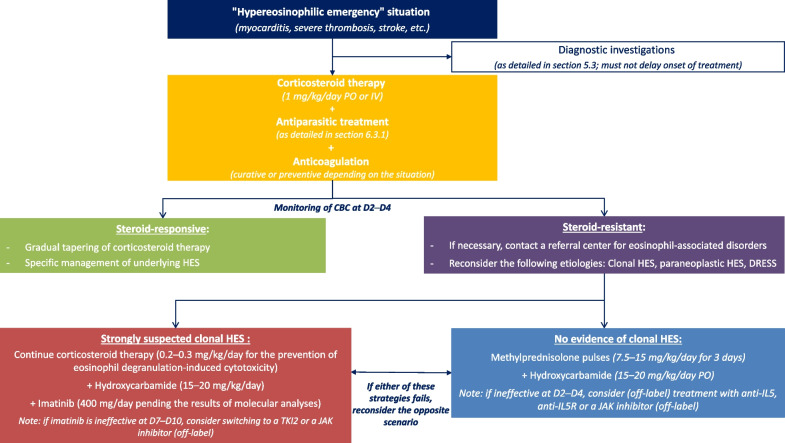
Proposed algorithm for the management of emergency situations related to HE. Abbreviations: DRESS, drug reaction with eosinophilia and systemic symptoms; HES, hypereosinophilic syndrome; IL, interleukin; IV, intravenous; PO, per os; TKI2, second-generation tyrosine kinase inhibitor

In the context of blood HE, some specific eosinophilic-related organ manifestations can be organ or life-threatening and thus require immediate treatment intensification (as detailed in Fig. 3). These mainly include cardiac manifestations (*e.g.,* eosinophilic myocarditis, intracavitary thrombosis with peripheral or cerebral embolic events, coronary artery spasm, acute heart failure), respiratory disorders (severe acute asthma or hypoxic lung disease), central nervous system manifestations (typically strokes of bilateral watershed distribution) and thrombotic (either arterial or venous) events [37–41]. Contrariwise, in the absence of overt clinical manifestations, very high eosinophil counts (e.g., > 10 × 10^9^/L) are not per se an indication for emergency initiation of treatment.

In these emergency situations, while the etiologic workup is also urgent, it should never delay the initiation of treatment. Thus, whatever the underlying cause of HE (reactive, idiopathic, clonal, but most often not determined at this stage), the initial management of severe eosinophilic vascular, respiratory, cardiac or neurological involvement that is life-threatening or can cause irreversible loss of function is based on corticosteroid therapy (prednisone: 1 mg/kg/day, preceded by intravenous pulses of methylprednisolone 15 mg/kg for 1–3 days, depending on the clinical picture), in combination with antiparasitic treatment (as detailed in “Antiparasitic treatment” Section).

In most cases, corticosteroids rapidly lower or normalize blood and tissue eosinophilia. In reactive HES, treatment should also include the management of the underlying disease or cessation of the culprit drug. In clonal eosinophilia, although corticosteroids generally do not normalize absolute eosinophil counts, they may reduce eosinophil activation and lower their cytotoxic potential pending a precise molecular diagnosis and onset of a specific treatment (as detailed in “Management of *FIP1L1::PDGFRA*–positive chronic eosinophilic leukemia” and “Management of non-F/P-associated clonal HES” Sections).

In case of steroid-resistant HES (defined as the absence of clinical and laboratory response after 3 days of corticosteroid therapy), the main etiologies to be investigated are as follow:

- Clonal HES (including F/P + chronic eosinophilic leukemia)
- Paraneoplastic eosinophilia
  - Hodgkin's lymphoma or high-grade peripheral T-cell lymphoma (especially angioimmunoblastic T-cell lymphoma)
  - Solid neoplasms (particularly bronchopulmonary carcinoma and colorectal or prostate adenocarcinoma).
- Rare corticosteroid-resistant DRESS

In rare cases of life-threatening clinically and biologically corticosteroid-refractory HES, it is recommended to contact a member of the CEREO network to discuss how to proceed with gradual and rapid treatment escalation (pending the etiological workup), which may include the use (sometimes sequentially) of:

- Hydroxycarbamide (15–20 mg/kg/day).
- Imatinib (at a dosage of 400 mg/day pending molecular testing)
- JAK inhibitors (including ruxolitinib) if clonal HES is strongly suspected and imatinib 400 mg/day has failed.
- Although the usefulness of biologic therapies such as anti-IL5 and anti-IL5R has only seldom been assessed in the context of emergency, their potential usefulness cannot be ruled out [112].
- As a last resort, alemtuzumab (the rationale being that CD52 is expressed on the surface of eosinophils, and the potential rapid onset of action of this treatment) [113].
- Conversely, while older studies had described the use of leukapheresis in this situation, it has become rare since the advent of targeted therapies [114].

Finally, in case of massive eosinophilia and/or manifestations related to micro/macrovascular complications of HE, curative anticoagulation may be recommended in the acute phase until HE subsides.

#### Vascular manifestations and HE

Eosinophils have significant proaggregatory and procoagulant properties, which may lead to (venous or arterial) thrombosis, vasculopathy (sometimes mimicking thromboangiitis obliterans), eosinophilic vasculitis (outside the context of EGPA or periarteritis nodosa) or even coronary artery spasm in HES [41, 59, 60]. There are currently no expert recommendations for the management of vascular thrombosis secondary to HE.

##### In the acute phase

In case of severe thrombosis, the "etiological" management of HE is similar to the procedure for the management of emergency situations involving HE detailed in “Follow-up of F/P + HE/HES” section. Given the ability of corticosteroids to induce rapid normalization of the CBC in most situations, the initiation of systemic corticosteroid therapy at the acute phase should be considered on a case-by-case basis. To date, there is no scientific data favoring either vitamin K antagonists or direct anticoagulants.

##### Long-term

The optimal duration of anticoagulant therapy is unknown. A multivariate analysis performed in a retrospective study of 54 patients with venous thromboembolism secondary to HE (regardless of the underlying etiology, but in the absence of any other major risk factor for thrombophilia) reported that persistent eosinophilia (> 0.5 × 10^9^/L) was the only factor associated with both the recurrence of venous thromboembolism as well as the occurrence of arterial thrombosis, suggesting that long-term normalization of the CBC (through effective control of the underlying disease) is crucial. Also, the fact that in this study the recurrence of venous thromboembolism occurred in only 1 of 16 patients who discontinued anticoagulants despite long-term normalization of the CBC also suggests that anticoagulant therapy may be discontinued on a case-by-case basis in patients with well-controlled HES (pending the fact that other underlying risk factors for thrombophilia and the anatomic site of thrombosis are also considered) [40]. Finally, given the high rates of arterial thrombosis reported in patients with venous thrombosis, non-specific management of cardiovascular risk factors also seems advisable in this situation.

#### Pregnancy

In some patients, pregnancy and the postpartum period seem to be risk periods for initial episodes or relapses of HE/HES. However, to date, there is no convincing evidence of specific eosinophil-related obstetric (notably placental vascular pathology, thrombosis, etc.) or fetal complications.

In practice, and pending further data:

- HE or HES is not a contraindication to pregnancy.
- Patients with HE/HES should have access to standard delivery, anesthesia, and breastfeeding options.
- The decision to conceive should ideally be discussed in advance with the HES specialist, considering the severity of organ involvement, any history of thrombosis associated with HES and medication (particularly TKI, hydroxycarbamide and anti-IL5), which are potentially advised against or contraindicated in pregnancy (and sometimes require a washout period prior to pregnancy, including imatinib in men). For anti-IL5 and anti-IL5R biologics, in the absence of substantial data supporting the safety or toxicity of these compounds in pregnancy [115, 116], the decision may be discussed on a case-by-case basis in a multidisciplinary team meeting.
- In the absence of a history of HE or exacerbation of HES during pregnancy (or postpartum) in a patient followed up for HES, a CBC should be performed (i) in preparation for the planned pregnancy (enhanced monitoring is advisable in case of HE); (ii) at diagnosis, and monthly during pregnancy; (iii) during the postpartum period.
- The following are recommended in patients with a history of exacerbation of HES and/or increased eosinophil counts during pregnancy or postpartum:
  - Close collaborative follow-up (involving the obstetrician-gynecologist and referring physician).
  - Monthly monitoring of the CBC during pregnancy and postpartum as well as to educate the patient on the symptoms that should prompt her to consult her general practitioner and perform a CBC whenever in doubt.
  - No cessation of ongoing corticosteroid therapy before or during pregnancy, but maintenance of a low dose or the minimum effective dose known to the patient during pregnancy and postpartum (3 months), before considering gradual tapering with laboratory monitoring (monthly CBC).
  - Multidisciplinary team meeting to discuss preventive anticoagulation (LMWH at prophylactic doses) in case of significant and/or rapidly progressive HE.

#### Children

Some specific factors also need to be considered in this population. Physical growth and pubertal development should be monitored, as well as the psychological impact of the disease and its treatment. Attempts should be made to reduce corticosteroid exposure as much as possible to limit potential steroids-related side-effects e.g., by not exceeding the maximum dose of 2 mg/kg/day of prednisone or equivalent, by trying to reduce the dose whenever the clinical situation and laboratory results allow so, or by considering a rapid switch to a second-line steroid-sparing treatment. In addition, peginterferon alfa-2a, mepolizumab and benralizumab have not been assessed in children under 3, 6, and 12 years of age, respectively. For imatinib, the dose should be adjusted based on body surface area, and close monitoring of physical growth in treated children is recommended (there have been reports of stunted growth in children and preadolescents receiving imatinib). Although not currently published, real-life data suggest that ciclosporin or JAK inhibitors (e.g., ruxolitinib) may also be useful in the context of pediatric HES. In adolescence, early contact with adult medical care teams, as well as collaborative management, is advisable to facilitate the transition to adulthood.

### Other drugs that can be used to treat HES

Given the variety of clinical manifestations that may be encountered in HES, various adjunctive symptomatic treatments may be offered as appropriate. These include (non-exclusive list):

- Antiplatelet agents in case of HES with a vascular phenotype requiring the management of cardiovascular risk factors.
- Antidepressant(s) in case of psychological complications of chronic HES, and/or neurogenic pain.
- Oral antidiabetics and/or insulin therapy in case of steroid-induced diabetes.
- Antihistamines for the treatment of urticaria.
- Bisphosphonates (when indicated) for the prevention of steroid-induced osteoporosis.
- Contraceptives: especially during treatment with hydroxycarbamide, TKI and anti-IL5 therapies.
- Beta-blockers, angiotensin converting enzyme inhibitors angiotensin receptor blockers, SGLT2 inhibitors and/or diuretics (in case of chronic heart failure).
- Asthma treatments (including short- and long-acting beta-2 agonists, anticholinergics and antileukotrienes).
- Annual influenza vaccination, prime-boost pneumococcal vaccination (13-valent conjugate vaccine followed at least 8 weeks later by a 23-valent polysaccharide vaccine) in patients receiving corticosteroids and/or immunomodulators. Vaccination against SARS-CoV-2 is also recommended.

### Surgical treatments

#### Use of cardiac surgery

Since the advent of targeted therapies (TKI, biologic therapies targeting IL-5), end-stage heart failure (endomyocardial fibrosis, valvular insufficiency, etc.) secondary to poorly controlled chronic HE has become exceptional. Nevertheless, when it is diagnosed at a late stage and the patient fails to recover despite prolonged normalization of the CBC and well-managed treatment of cardiac symptoms, cardiac surgery (valve replacement and/or endocardial decortication, or even heart transplantation as a last resort) is an option that can be discussed on a case-by-case basis in the event of refractory heart failure [38, 117]. HES that is amenable to targeted therapy (e.g., with imatinib) leading to long-term remission (especially cases involving *PDGFRA* or *PDGFRB* rearrangements) is not a contraindication to heart transplantation. In this case, specific treatment of HES should be continued over the long-term after transplantation to prevent recurrence of HES in the transplant [118].

#### Use of ENT surgery

In case of nasal polyposis refractory to topical and systemic corticosteroid therapy, it is recommended to seek specialist advice to discuss the indications for and modalities of surgical management (polypectomy or ethmoidectomy), if appropriate.

### Paramedical support

#### Patient support group

The primary care physician or specialist may encourage the patient to contact a patient support group association (if available).

In France, APIMEO (*Association Pour l'Information sur les Maladies à Eosinophiles*—Association for Information on Eosinophilic Diseases) is a patient support group consisting of patients and their relatives, whose main tasks are to:

- Bring patients and their relatives out of isolation.
- Contribute to better management of the disease.
- Facilitate the sharing of experiences and information between patients at dedicated events.
- Promote and participate in therapeutic educational activities.
- Disseminate information validated by the scientific committee.
- Help promote research.
- Work together with public authorities to improve patient care and quality of life.
- Participate in mixed working groups (doctors/patients) to share experiences and facilitate the dissemination of information to improve patient quality of life.
- Represent patients in various healthcare institutions.
- Initiate local or national events to spread awareness about eosinophilic diseases.

#### Therapeutic patient education

##### aDefinition

Therapeutic patient education (TPE) is offered to all people with chronic diseases who may feel the need for it. This approach is centered on patients and their immediate circles and is integrated into the care process. It aims to increase their knowledge about their condition and eventually to improve their independence and quality of life.

##### Practicalities for HES

The French Healthcare Network for Rare Immune-Hematological Diseases *(MaRIH, filière de Santé Maladies rares immuno-hématologiques*) and CEREO teamed up to launch a TPE program focused on HES, organ-specific eosinophilic disorders and their treatment, entitled ‘*Les éosinos … kézako*' ("Eos-what?"). The program, which has been accredited by both the Regional Health Agencies of Ile-de-France and Hauts-de-France, is aimed at patients over 16 years of age (and their families) managed in a CEREO facility.

It is led by a multidisciplinary team specifically trained in TPE, including health professionals (internists, pulmonologists, gastroenterologists, dermatologists, and nurses), social workers, clinical research assistants, management assistants, nutritionists, and patients with expertise, in partnership with APIMEO, a patient association for eosinophilic disorders.

It takes the form of monthly workshops (lasting 1.5–2 h each), either face-to-face or remote by videoconference.

The program is divided into different phases:

1. A shared educational assessment (in person or by phone) to better understand the patient's needs, expectations, concerns, beliefs, and plans.
2. For each patient, a personalized course is defined involving participation in some or all of the 8 educational workshops offered as part of the program.
3. Delivery of TPE workshops, each dedicated to a specific topic (*e.g.* overview of eosinophil-associated diseases and their treatments, focus on respiratory, GI or skin manifestations, how to prevent steroids-induced side-effects…) and with a standardized structure and content.
4. Review and individual evaluation of the "skills" acquired by the patient at the end of the educational program, a summary of the patient’s course being sent to his General Practitioner.

#### Other

Depending on each person's situation, the following may be offered on a case-by-case basis (in a non-exhaustive manner) in situations related to HES and/or its treatment:

- Physical therapy: in case of motor or proprioceptive symptoms related to HES.
- Support from a nutritionist.
- Support from a psychotherapist.
- Support from a social worker.

Also, in case of progression to multiple disabilities (which remain exceptional in the context of HES), it may be necessary to adjust daily life, prescribe medical support devices and/or apply for social assistance from disability support organizations.

## Follow-up

### Aims

The objectives of follow-up are to:

- Monitor the effectiveness of treatment on the clinical manifestations of HES, which should be assessed based on absolute eosinophil counts.
- Screen for other organ dysfunctions potentially associated with persistent HE.
- Identify and treat exacerbations early.
- Identify and address potential factors that may contribute to poor treatment compliance.
- Prevent, identify and, if necessary, provide early management of potential treatment-related complications.
- Limit, identify and, if necessary, provide early management of disease sequelae.
- Assess the psychological, family, scholastic and social/professional impact of the disease and limit its consequences.
- Cover the transition from pediatric to adult medicine.

### Professionals involved

The consultations required during treatment depend on the baseline assessment and the clinical course. Follow-up is generally multidisciplinary and coordinated by a hospital physician.

It is carried out by the same professionals as those involved in the initial assessment, plus other allied health professionals and social assistance professionals, if necessary.

### Frequency and content of consultations

The monitoring frequency depends on the duration and severity of the disease. Two specialist consultations per year are usually sufficient when the disease is well controlled.

Monitoring consists of a complete physical examination and quarterly (or more frequent, if indicated) monitoring of CBC. If necessary, clinical and laboratory monitoring may be supplemented by other complementary examinations (radiological, endoscopic, etc.) depending on the clinical manifestations of HES or the type of HES (e.g., pulmonology consultation with lung function tests in case of asthma, chest CT in case of eosinophilic pneumonia, transthoracic echocardiogram in case of eosinophilic heart disease, etc.) or treatment (dermatological monitoring in case of treatment with hydroxycarbamide).

As the blood eosinophil count is a relatively reliable marker of the degree of tissue eosinophilia, it is not recommended to repeat invasive and/or expensive examinations if the CBC is well controlled. Similarly, if eosinophilic infiltration has been documented at least once in the affected organ and differential diagnoses have been ruled out, the clear correlation between eosinophil counts and clinical symptoms in most patients justifies not repeating invasive examinations (e.g., GI endoscopy).

However, in the event of persistent HE > 1.5 × 10^9^/L in patients not under treatment or in partial remission under treatment, regular heart examinations (troponin and BNP assays and TTE) are recommended (at frequent intervals in the first months following the onset of HE due to the high risk of organ complications during this period, and at longer intervals, e.g., annually thereafter) are recommended even in the absence of any symptoms due to the risk of pauci-symptomatic disease.

To date, there is no validated quality of life scale for HES, but scales designed for the assessment of specific symptoms (such as the Asthma Control Test or the Sino-Nasal Outcome Test) may be used if necessary.

### Follow-up specificities according to the disease subtype

#### Follow-up of F/P + HE/HES

##### After initiation of imatinib,

Patients are initially closely monitored, with:

- Weekly CBCs until levels return to normal (which typically occurs within the first month) and then every 3 months, plus serum electrolytes and complete liver function tests).
- Molecular testing for the *FIP1L1::PDGFRA* transcript: at 3 months after initiation of imatinib, then every 6 months for 2 years, then annually thereafter).

##### If imatinib is discontinued

If imatinib treatment is discontinued (ideally after at least 5 years of treatment), in the same manner as when it is discontinued in chronic myeloid leukemia, close molecular monitoring (every 3 months for the first year and every 6 months thereafter) is required in the long-term (even in the absence of laboratory evidence of HE, to avoid missing a molecular relapse).

##### Various other considerations

Imatinib is generally well tolerated, with an incidence of less than 5% of grade 3 or 4 adverse events in the French case series of F/P + chronic eosinophilic leukemia. The main toxic effects are hepatic, muscular, and hematologic. Superficial edema (particularly periorbital), non-specific GI symptoms, hypopigmentation and photosensitization may also occur.

#### Follow-up of non-F/P-associated clonal HE/HES

##### Clonal HES related to a tyrosine kinase rearrangement (other than F/P)

Because of the rarity of cases, the clinical/molecular follow-up of this type of clonal HES is not standardized. For practical purposes, the modalities described for F/P + chronic eosinophilic leukemia may be used. Regular cytogenetic follow-up is therefore required, especially in the absence of a specific molecular test.

##### Chronic eosinophilic leukemia–NOS

Due to the rarity of CEL-NOS, the modalities of follow-up have not been standardized. Since there is a potential for progression to acute myeloid leukemia, regular monitoring (at least annually and/or more frequently in the event of cytopenia/increased blast count suggestive of progression) of cytogenetic and molecular data (including by NGS-based gene panel tests) is recommended.

##### Other considerations

Hydroxycarbamide may be associated with hematologic (cytopenia), dermatologic (oral ulcerations, non-melanocytic skin tumors, hyperpigmentation, hair loss) and hepatic (cytolysis) adverse effects that call for regular clinical and laboratory monitoring (e.g., every 2 weeks for 2 months after initiation of treatment, then monthly for 3 months, and then every 3 months thereafter).

The tolerability of TKI other than imatinib is generally good, although there are some issues worth noting. Besides the (non-specific) hepatic, muscular and hematologic toxicity of TKI:

- Nilotinib can be associated with dermatologic (rash, xerosis, pruritus), hepatic (usually moderate cytolysis, but also hyperlipidemia and/or free hyperbilirubinemia) and cardiovascular adverse reactions (arterial obstruction).
- Ponatinib is associated with frequent cardiovascular adverse reactions (including hypertension and arterial obstruction) and should be used with caution in patients with a history of cardiovascular disease. An assessment of cardiovascular risk (and the implementation of preventive measures) prior to initiating treatment is also advisable.
- Treatment with dasatinib can be associated with GI bleeding (although this is not a formal contraindication, caution should be exercised in patients with a history of gastritis or ulcers and/or in patients with thrombocytopenia or receiving antiplatelet/anticoagulant therapy) and pleural effusion (which is a contraindication to continuation of treatment).

#### Follow-up of lymphocytic HE/HES

Although rare (about 5–10% of patients), screening for transformation into high-grade T-cell lymphoma should be done at each visit, especially in case of clinical (e.g., general symptoms, new-onset lymphadenopathy) and/or biological ones (e.g., laboratory evidence of inflammation, cytopenia, etc.) suggestive of the diagnosis. When in doubt, a new PET scan can be performed to guide a lymph node biopsy. In ambiguous cases, a bone marrow biopsy may also be performed. Some discriminating criteria are suggested in Box 5 and in case of doubt, the advice of a trained pathologist should be sought.

While data are limited (due to the rarity of lymphocytic HES), some authors have suggested that there might be some degree of correlation between the percentage of abnormal T cells (especially CD3− CD4+) and the clinical course (i.e., an increased percentage of abnormal T cells could be associated with poorer control of clinical manifestations). However, there is no evidence to date suggesting that increased abnormal T-cell populations may be an early warning sign of transformation into high-grade T-cell lymphoma. In general, until further data become available, annual monitoring of T-cell phenotype is not recommended routinely.

## Conclusion

Here, we provide practical recommendations for both the step-by step diagnostic workup of eosinophilia (whether unexplained or within specific contexts) as well as the management and follow-up of the full spectrum of eosinophil-associated disorders (including clonal, reactive, lymphocytic and idiopathic HES, as well as single-organ diseases). Didactic prescription summaries intended to facilitate the prescription of eosinophil-targeted drugs are also provided, as are practical diagnostic and therapeutic algorithms. Although this expert consensus is likely to inform and assist health professionals in the diagnostic and/or therapeutic management of patients with HE/HES, it is—due to the wide range of clinical conditions grouped under the term of HES—not a substitute for expert opinion on a case-by-case basis. Further updates will be mandatory as new validated information emerges.

## Supplementary Information

**Additional file 1:** Summary intended for general practitioners

**Additional file 2:** List of referral centers of the French Eosinophil Network

**Additional file 3:** Main cosmopolitan parasitic infections

**Additional file 4:** Main cytogenetic abnormalities that can lead to clonal hypereosinophilia

**Additional file 5:** Screening strategy for organ involvement in patients with hypereosinophilia

## Data Availability

Not applicable.

## Abbreviations

CBC: Complete blood count
CCL: Chemokine ligand
CCR: C-C chemokine receptor
DRESS: Drug reaction with eosinophilia and systemic symptoms
ECP: Eosinophil cationic protein
EGPA: Eosinophilic granulomatosis with polyangiitis
EPX: Eosinophil peroxidase
FGFR: Fibroblast growth factor receptor
FIP1L1: Factor interacting with PAPOLA and CPSF1
FISH: Fluorescence in situ hybridization
GI: Gastrointestinal
HE: Hypereosinophilia
HES: Hypereosinophilic syndrome
HEus: HE of undetermined significance
ICOG-Eo: International cooperative working group on eosinophil disorders
IL: Interleukin
ILC2: Type 2 innate lymphoid cells
JAK: Janus kinase
KIT: Mast/stem cell growth factor receptor
MBP: Major basic protein
NGS: Next-generation sequencing
NOS: Not otherwise specified
PDGFRA: Platelet-derived growth factor receptor A
PDGFRB: Platelet-derived growth factor receptor B
PCR: Polymerase chain reaction
PET: Positron emission tomography
STAT: Signal transducer and activator of transcription
TKI: Tyrosine kinase inhibitor
TPE: Therapeutic patient education
