# Supplementary material for: French guidelines for the etiological workup of eosinophilia and the management of hypereosinophilic syndromes
Source: Orphanet J Rare Dis. 2023 Apr 30;18:100. doi: 10.1186/s13023-023-02696-4 (PMC10148979; doi:10.1186/s13023-023-02696-4)
Supplement: Supplementary file 1 — Additional file 1: Summary intended for general practitioners [file 13023_2023_2696_MOESM1_ESM.docx]

**Appendix 1 – Summary intended for general practitioners**

This summary was prepared based on the National Diagnostic and Care Protocol (NDCP) – Hypereosinophilia and Hypereosinophilic Syndromes available at [www.has-sante.fr](http://www.has-sante.fr) and www.cereo.fr.

Hypereosinophilic syndrome (HES) is defined as blood hypereosinophilia (HE) ≥ 1.5 x10^9^/L and persistent tissue hypereosinophilia (>1 month) associated with tissue damage (of any kind) due to eosinophil toxicity.

It is a heterogeneous condition that includes:

- **Clonal** (or neoplastic) **HES**, including chronic eosinophilic leukemia associated with the 4q12 deletion responsible for the *FIP1L1*::*PDGFRA* (*F/P*) fusion.
- **Reactive**(secondary) **HES** caused by parasitic infections, medication or inflammatory or neoplastic diseases. Reactive HES also includes **lymphocytic HES** which is due to low-grade proliferation of T cells bearing an abnormal phenotype (usually CD3-CD4+).
- **Idiopathic HES.** Despite extensive investigations, the etiology of up to 3/4 of HES cases remains unknown.

The conditions covered in this NDCP include all types of HES, including single-organ eosinophilic disorders (except for eosinophilic esophagitis and hypereosinophilic asthma).

## Initial assessment

The spectrum of HES includes heterogeneous disorders whose management requires multidisciplinary assessment tailored to each patient. Depending on the complexity of the case, this assessment is performed by:

- General practitioners or pediatricians.
- Specialists involved in the management of the various clinical manifestations.
- Reference centers (in France, centers working in partnership with the French National Reference Center for HES (CEREO) (**Appendix 1**).

The aim of the initial assessment is to:

- Investigate the main "usual" causes of HE/HES (parasitic infection, drugs, etc.) using a specific, individual diagnostic approach and perform complementary investigations in case of unexplained HE (specialist consultation).
- Screen for the main types of eosinophil-induced organ dysfunction and specify the severity of organ damage, if applicable.
- Rule out differential diagnoses (not all cases of organ dysfunction associated with HE are necessarily caused by HE itself).
- Establish therapeutic indications, considering any comorbidities likely to affect prognosis, treatment tolerability.

Generally:

- The diagnosis of HES should be considered in any case of blood HE ≥ 1.5 x10^9^/L and/or tissue HE associated with organ dysfunction. The clinical manifestations are diverse, but most commonly involve the skin, respiratory and gastrointestinal systems; cardiac and thrombotic involvements are less common but can be life-threatening.
- Any patient with HE x10^9^/L should undergo an etiological workup and a minimal assessment, as detailed in **Box 4**. Further investigations (summarized in **Box 9**) are carried out depending on the findings of the physical examination findings and the type of HES (as described in section 5.3.4).
- Although there are some predominant symptoms (e.g., cardiac involvement in clonal HES, angioedema and articular manifestations in lymphocytic HES), all clinical manifestations of HES are possible, regardless of the underlying pathophysiological mechanism of HE.
- Since the definition of HES implies eosinophilic infiltration of involved tissues, histologic or cytologic confirmation (e.g., by bronchoalveolar lavage) is always desirable. However, if no eosinophilic infiltration is detected (e.g., high-risk biopsy procedure, pre-existing treatment with systemic corticosteroids etc.) HES-related organ involvement can also be diagnosed by default when all the following criteria are met: Blood HE, organ involvement consistent with HES (including the lack of any differential diagnosis) and parallel course of organ involvement and blood HE.

## Therapeutic management

Therapeutic management is generally multidisciplinary and may be undertaken in partnership with a CEREO reference center and/or center of expertise **(Appendix 1)**.

The choice of treatment, treatment modalities (short-term vs. prolonged) and their goals (normalization of the complete blood count (CBC) vs. control of symptoms while tolerating a certain level of persistent eosinophilia) depends on the type of HES, the severity of the disease, the risk of relapse, and the patient's condition and risk factors (age, possible comorbidities). A general algorithm for the management of HES is provided in **Figure 1.**

The treatment of HES relies mainly on the following:

- For clonal HES: typically tyrosine kinase inhibitors (particularly imatinib) and hydroxycarbamide.
- For reactive and idiopathic HES: corticosteroids (topical and/or systemic), peginterferon alfa-2a, hydroxycarbamide and mepolizumab.

Following two clinical drug trials that demonstrated the superiority of mepolizumab (a humanized IgG1 kappa monoclonal antibody targeting IL-5) vs. placebo (both in terms of reduction of the number of clinical relapses and steroid sparing effect), this drug was approved by the EMA in November 2021 and by November 1^rst^ 2022 is now reimbursed by the French National Health Insurance.

In case of failure of standard treatments, cases can be referred for further discussion in the monthly national multidisciplinary meetings held by the CEREO team.

Depending on the clinical picture and the HES subtype, other treatments may also be considered. The latter may include:

- Curative anticoagulant therapy in case of HE-related thromboembolism or cardiac disease at risk of embolism; preventive anticoagulant therapy.
- Prophylaxis of certain iatrogenic complications (including infections, glucocorticoid-induced osteoporosis).
- Surgical procedures (in case of advanced endomyocardial fibrosis, for example).

Some severe clinical manifestations, including cardiac (myocarditis, intracavitary thrombosis with peripheral or cerebral embolic events, coronary artery spasm), acute respiratory distress (severe acute asthma, hypoxic lung disease), or thrombotic events (venous or arterial) require urgent treatment with a combination of different drug classes with anti-eosinophilic activity used sequentially (as detailed in **Figure 3**) before the findings of the etiological HE workup become available.

Information and therapeutic education of patients and their immediate relatives is also encouraged.

Lastly, all health professionals and patients should be made aware of the existence of patient associations.

## Pregnancy

Although the underlying mechanisms remain unclear, cases of HE and HES are sometimes diagnosed during pregnancy and postpartum, with possible recurrence in subsequent pregnancies. The initial diagnostic procedure should be the same as for any case of HE or HES and include the investigation of organ involvement (e.g., cardiac involvement). As a general rule, an opinion should be sought from a center of expertise in the event of unexplained HE or organ complications during pregnancy.

Special attention should be paid to potentially fetotoxic or teratogenic drug treatments for HES, such as hydroxycarbamide and imatinib. In men who are planning to father a child, paternal exposure to certain drugs may also temporarily contraindicate pregnancy.

## Children

HE and HES are sometimes diagnosed in children. The initial diagnostic procedure should be the same as for any case of HE or HES and include the investigation of organ involvement (e.g., cardiac involvement). In the event of unexplained HE, a hematologic malignancy (acute lymphoblastic or myeloid leukemia, Hodgkin's lymphoma) should be ruled out in case of severe HE (e.g., >10 x10^9^/L) and/or associated CBC abnormalities, splenomegaly, or initial steroid resistance. Exceptionally, certain genetic factors may be responsible for HE or HES in infants, including some types of primary immunodeficiency. Given the underlying causes and chronic nature of HES, special attention should be paid to the potential impact of the disease or treatment on child development, nutrition, and growth/puberty.

## Follow-up

The objectives of follow-up are to:

- Monitor the effectiveness of HES treatment as measured by the course of the symptoms and the absolute eosinophil count.
- Screen for any other organ dysfunction associated with persistent HE.
- Identify and treat relapses early, whether they involve pre-existing or new-onset organ dysfunction.
- Assess and attempt to correct possible reasons for poor treatment compliance.
- Limit, identify and, if necessary, provide early management of potential treatment-related complications, including effects on physical growth (height and weight) in children.
- Limit, identify and, if necessary, provide early management of disease sequelae.
- Assess the psychological, family, scholastic and social/professional impact of the disease and limit its consequences.

The monitoring frequency should be based on the duration and severity of the disease. Two specialist consultations per year are usually sufficient when the disease is under control.

Monitoring consists of a complete physical examination and quarterly (or more frequent, if indicated) monitoring of CBC. If necessary, physical and laboratory monitoring may be supplemented by other complementary examinations (radiological, endoscopic, etc.) depending on the clinical manifestations of HES or the type of HES (including regular molecular and/or cytogenetic monitoring depending on the existence and nature of gene rearrangement in case of clonal HE).

As the blood eosinophil count is a relatively reliable marker of disease progression, it is not recommended to repeat invasive examinations or to increase maintenance treatment in the absence of blood HE.

In case of persistent blood HE >1.5 x10^9^/L (in the context of non-treatment or partial remission under treatment), annual cardiac function tests (troponin and BNP assays and transthoracic cardiac echocardiography) are recommended, even in the absence of symptoms, because of the risk of pauci-symptomatic disease. While cardiac MRI is not routinely performed, it should be considered on a case-by-case basis depending on clinical, laboratory and/or echocardiographic findings, as well as in patients with clonal HE/HES (due to the higher risk of endomyocardial fibrosis).
