# Supplementary material for: French guidelines for the etiological workup of eosinophilia and the management of hypereosinophilic syndromes
Source: Orphanet J Rare Dis. 2023 Apr 30;18:100. doi: 10.1186/s13023-023-02696-4 (PMC10148979; doi:10.1186/s13023-023-02696-4)
Supplement: Supplementary file 2 — Additional file 2: List of referral centers of the French Eosinophil Network [file 13023_2023_2696_MOESM2_ESM.docx]

**Appendix 2 – List of Referral centers of the French Eosinophil Network (CEREO)**

| **CENTER** | **PURPOSE** | **PRINCIPAL INVESTIGATOR** |
| --- | --- | --- |
| Foch | Coordinating center | Dr. Matthieu Groh;  Prof. Jean-Emmanuel Kahn |
| CHU de Lille | Co-coordinating center | Dr. Guillaume Lefèvre |
| CHU d'Annecy | Center of expertise | Dr. Antoine Baudet |
| CHU de Bordeaux – Hôpital Haut-Lévêque | Center of expertise | Prof. Jean-François Viallard |
| CHU de Clermont-Ferrand | Center of expertise | Dr. Ludovic Trefond |
| CHU de Dijon | Center of expertise | Prof. Bernard Bonnotte |
| CHU de La Réunion | Center of expertise | Dr. Catherine Mohr |
| APHM – Hôpital de la Timone Marseille | Center of expertise | Prof. Nicolas Schleinitz |
| CHU de Montpellier | Center of expertise | Prof. Philippe Guilpain |
| CHU de Nantes – Hôtel Dieu | Center of expertise | Prof. Antoine Néel |
| CH de Perpignan | Center of expertise | Dr. Rodérau Outh |
| CHRU de Strasbourg – Hôpital Civil | Center of expertise | Dr. Aurélien Guffroy;  Prof. Thierry Martin |
| APHP – Ambroise Paré | Participant in the COHESION cohort* | Prof. Jean-Emmanuel Kahn |
| APHP – Avicenne | Participant in the COHESION cohort* | Prof. Sébastien Abad |
| APHP – Bichat | Participant in the COHESION cohort* | Dr. Antoine Dossier |
| APHP – Jean Verdier | Participant in the COHESION cohort* | Dr. Laurent Gilardin |
| APHP – Lariboisière | Participant in the COHESION cohort* | Prof. Damien Sène |
| APHP – Mondor | Participant in the COHESION cohort* | Dr. Nicolas Limal |
| APHP – Necker | Participant in the COHESION cohort* | Prof. Felipe Suarez |
| APHP – St. Antoine | Participant in the COHESION cohort* | Dr. Noémie Abisror |
| APHP – St Louis | Participant in the COHESION cohort* | Prof. Adèle de Masson |
| APHP – Tenon | Participant in the COHESION cohort* | Prof. Sophie Georgin-Lavialle |
| CH d'Amiens | Participant in the COHESION cohort* | Dr. Clément Gourguechon |
| CHU d'Angers | Participant in the COHESION cohort* | Prof. Mathilde Hunault |
| CH d'Arras | Participant in the COHESION cohort* | Dr. Mathieu Jouvray |
| CH d'Avignon | Participant in the COHESION cohort* | Dr. Borhane Slama |
| CH de Bayonne | Participant in the COHESION cohort* | Dr. Irène Machelart |
| CHRU de Besançon | Participant in the COHESION cohort* | Dr. Sébastien Humbert |
| CH de Blois | Participant in the COHESION cohort* | Dr. Abderrazak El Yamani |
| CHU de Bordeaux (Unicancer) | Participant in the COHESION cohort* | Dr. Gabriel Etienne |
| CHU de Caen | Participant in the COHESION cohort* | Prof. Achille Aouba |
| CH de Dax | Participant in the COHESION cohort* | Dr. François Lifermann |
| GH Diaconesses Croix St-Simon | Participant in the COHESION cohort* | Dr. Benjamin Subran |
| CH de Dunkerque | Participant in the COHESION cohort* | Dr. Amélie Leurs |
| CHU de Grenoble | Participant in the COHESION cohort* | Dr. Maxime Lugosi |
| CH Le Mans | Participant in the COHESION cohort* | Dr. Pierre Lozach |
| CHU de Limoges | Participant in the COHESION cohort* | Dr. Holy Bezanahary |
| CHU de Lyon – HCL | Participant in the COHESION cohort* | Prof. Vincent Cottin |
| CH de Melun | Participant in the COHESION cohort* | Dr. Nabil Belfeki |
| CH Mont de Marsan | Participant in the COHESION cohort* | Dr. Arnaud Saint Lezer |
| CHU de Nancy | Participant in the COHESION cohort* | Dr. Thomas Moulinet |
| CHU de Nice | Participant in the COHESION cohort* | Dr. Viviane Queyrel |
| CHU de Poitiers | Participant in the COHESION cohort* | Dr. Mathieu Puyade |
| CHI de Poissy-St-Germain-en-Laye | Participant in the COHESION cohort* | Dr. Azzedine Bouderbala |
| CHU de Rouen | Participant in the COHESION cohort* | Dr. Maximilien Grall |
| CHU de Saint-Etienne | Participant in the COHESION cohort* | Prof. Pascal Cathébras |
| CH de St. Quentin | Participant in the COHESION cohort* | Dr. Sara Melboucy-Belkhir |
| CH de Toulon (Ste Anne) | Participant in the COHESION cohort* | Dr. Jean-Sébastien Blade |
| CHU de Toulouse (Purpan) | Participant in the COHESION cohort* | Dr. Guillaume Moulis |
| CHU de Toulouse (Oncopôle) | Participant in the COHESION cohort* | Dr. Suzanne Tavitian |
| CH de Valenciennes | Participant in the COHESION cohort* | Dr. Thomas Quemeneur |
| CH de Vannes | Participant in the COHESION cohort* | Dr. Yoann Crabol |
| CH de Versailles | Participant in the COHESION cohort* | Prof. Philippe Rousselot |
| CH de Vesoul | Participant in the COHESION cohort* | Dr. Cécile Golden |

*National cohort of patients with HE or HES; for more information, please visit http://www.cereo.fr/website/cohorte_cohesion_&400&39.html
