# Supplementary material for: French guidelines for the etiological workup of eosinophilia and the management of hypereosinophilic syndromes
Source: Orphanet J Rare Dis. 2023 Apr 30;18:100. doi: 10.1186/s13023-023-02696-4 (PMC10148979; doi:10.1186/s13023-023-02696-4)
Supplement: Supplementary file 3 — Additional file 3: Main cosmopolitan parasitic infections [file 13023_2023_2696_MOESM3_ESM.docx]

**Appendix 3 – Main cosmopolitan parasitic infections**

| Parasite | Mode of infection | Hypereosinophilia (HE) | Diagnostic confirmation method |
| --- | --- | --- | --- |
| Anisakiasis  (Anisakis sp) | Ingestion of contaminated raw fish (herring) | Inconsistent HE, sometimes massive | EGD |
| Ascariasis  (Ascaris lumbricoides) | Ingestion of contaminated food | Elevated HE, which may be normal during the emergence phase | Serology (first-time infection)  O&P (emergence phase) |
| Diphyllobothriasis  (Diphyllobothrium latum) | Ingestion of raw freshwater fish | Moderate HE | O&P |
| Liver fluke infection  (Fasciola hepatica) | Ingestion of contaminated plants (cress) | High HE | Serology  O&P |
| Alveolar echinococcosis  (Echinococcus multilocularis) | Ingestion of contaminated plants (Eastern France) | HE <1500/mm^3^ | Serology |
| Hydatid disease  (Echinococcus granulosus, dead-end host) | Contact with infected dogs or ingestion of contaminated food | HE <1500/mm^3^ (unless the hydatid cyst ruptures) | Serology |
| Pinworm infection  (Enterobius vermicularis) | Ingestion of eggs (self-infestation) | HE <1500/mm^3^ | O&P  Tape test |
| Taeniasis  (Taenia saginata or T. solium) | Consumption of undercooked meat (beef or pork) | HE <1500/mm^3^ | O&P  Serology |
| Toxocariasis  (Toxocara canis or T. cati) | Ingestion of contaminated food | Persistently high HE | ELISA test + WB |
| Trichinellosis  (Trichinella spiralis) | Consumption of game (wild boar), undercooked meat (pork, horse) | Persistently high HE | Serology |

ELISA: enzyme-linked immunosorbent assay; O&P: stool ova and parasite test; EGD: esophagogastroduodenoscopy; HE: hypereosinophilia; WB: Western blot
