# Supplementary material for: French guidelines for the etiological workup of eosinophilia and the management of hypereosinophilic syndromes
Source: Orphanet J Rare Dis. 2023 Apr 30;18:100. doi: 10.1186/s13023-023-02696-4 (PMC10148979; doi:10.1186/s13023-023-02696-4)
Supplement: Supplementary file 4 — Additional file 4: Main cytogenetic abnormalities that can lead to clonal hypereosinophilia [file 13023_2023_2696_MOESM4_ESM.docx]

**Appendix 4 – Main cytogenetic abnormalities that can lead to clonal hypereosinophilia***

| **Tyrosine kinase involved** | **Activation mechanism** | **Partner gene** |
| --- | --- | --- |
| **PDGFRA 4q12** | **Deletion**  Cryptic del(4)(q12)  **Translocation/insertion**  t(4;22)(q12;q11)  t(2;4)(p24;q12)  Complex  ins(9;4)(q33;q12q25)  t(4;12)(q12;p13) | *FIP1L1* 4q12  *BCR* 22q11  *STRN* 2p22  *KIF5B* 10p11  *CDK5RAP2* 9q33  *ETV6 (TEL)* 12p13 |
| **PDGFRB 5q32** | **Translocation**  t(5;12)(q32;p13)  t(5;15)(q32;q15)  t(5;14)(q32;q22)  t(5;17)(q32;p11)  t(1;5)(q21;q32)  t(5;10)(q32;q21)  t(5;7)(q32;q11)  t(5;17)(q32;p13)  t(5;14)(q32;q32) | *ETV6 (TEL)* 12p13  *TP53BP1* 15q15  *NIN* 14q22  *SPECC1-1* 17p11  *PDE4DIP* 1q21  *CCDC6* 10q21  *HIP1* 7q11  *RABEP1-5* 17p13  *TRIP11* 14q32 |
| **FGFR1 8p11** | **Translocation/insertion**  t(8;17)(p11;q11)  t(7;8)(q34;p11)  ins(12;8)(p11;p11-p22)  t(8;19)(p11;q13)  t(8;9)(p11;q33)  t(8;22)(p11;q11)  t(6;8)(q27;p11)  t(8;13)(p11;q12) | *MYO18A* 17q11  *TRIM24* q34  *FGFR1OP2* 12p11  *HERVK* 19q13  *CNTRL* 9q33  *BCR* 22q11  *FGFR1OP* 6q27  *ZMYM2* 13q12 |
| **JAK2 9p24** | **Translocation**  t(8;9)(p22;p24)  **Mutation** V617F | *PCM1* 8p22 |
| **FLT3 13q12** | **Translocation**  t(12;13)(p13;q12) | *ETV6 (TEL)* 12p13 |

* non-exhaustive list
