# Supplementary material for: French guidelines for the etiological workup of eosinophilia and the management of hypereosinophilic syndromes
Source: Orphanet J Rare Dis. 2023 Apr 30;18:100. doi: 10.1186/s13023-023-02696-4 (PMC10148979; doi:10.1186/s13023-023-02696-4)
Supplement: Supplementary file 5 — Additional file 5: Screening strategy for organ involvement in patients with hypereosinophilia [file 13023_2023_2696_MOESM5_ESM.docx]

**Appendix 5 – Screening strategy for organ involvement in patients with hypereosinophilia**

| **Organ** | **Possible clinical picture in HES** | **Clinical and laboratory findings necessary to rule out organ involvement** | **Investigations to confirm the diagnosis/assess the impact of HES** |
| --- | --- | --- | --- |
| **Heart** | Pericarditis, myocarditis, endomyocardial fibrosis, valvular disease, dilated cardiomyopathy, ventricular thrombus, rhythm and conduction disorders, etc. | Physical examination, ECG, troponin, BNP or NT-pro-BNP, normal TTE | MRI, coronary angiography or coronary CT angiography (*)  Endomyocardial biopsy (**) |
| **Skin** | Urticaria, eczematous lesions, angioedema, purpura, pruritus, maculopapular lesions | Absence of pruritus, skin lesions and angioedema | Biopsy |
| **Lung** | Eosinophilic pneumonia, pleurisy, hypereosinophilic asthma, eosinophilic bronchiolitis, etc. | Normal physical examination and chest CT | Chest CT scan  LFT  BAL  Bronchial biopsy (**) |
| **Liver, GI tract** | Eosinophilic esophagitis, eosinophilic gastritis, eosinophilic enteritis, eosinophilic colitis, eosinophilic pancreatitis, eosinophilic cholecystitis or cholangitis, eosinophilic ascites, eosinophilic hepatitis, etc. | Physical examination, complete liver panel, and abdominopelvic (AP) CT scan all normal | AP CT scan  EGD – Colonoscopy  MR cholangiogram, AP MRI  Abdominopelvic ultrasound  Ascites fluid cytology  GI biopsy  Liver biopsy (**) |
| **ENT** | Chronic rhinitis, chronic edematous rhinosinusitis, sinonasal polyposis | Physical examination with normal nasofibroscopy | CT scan of the sinuses |
| **Hematopoietic organs** | Lymphadenopathy, splenomegaly, etc. | Normal physical examination and CT scan of the chest, abdomen and pelvis | CT scan of the chest, abdomen, and pelvis, PET  Bone marrow biopsy, lymph node biopsy |
| **Peripheral nervous system** | Sensorimotor polyneuropathy, mononeuritis multiplex, isolated cranial nerve involvement, etc. | Normal physical examination | EMG  Neuromuscular biopsy (**) |
| **CNS** | Ischemic (or even embolic) stroke, cerebral vasculitis, spinal cord injury (inflammatory, ischemic, etc.), meningitis or meningoencephalitis, etc. | Normal physical examination | Brain MRI  Spinal MRI  Lumbar puncture |
| **Kidney** | Glomerulopathy, tubulointerstitial nephritis, etc. | Urine dipstick and renal function normal | Urine culture, urine protein levels  Renal biopsy |
| **Urinary tract** | Eosinophilic cystitis, eosinophilic ureteritis, etc. | Normal physical examination, normal urine dipstick test | CT urogram  Ultrasound  Pelvic MRI  Urine cytology  Cystoscopy and biopsy (**) |
| **Joints** | Inflammatory arthralgia, synovitis, etc. | Normal physical examination | Ultrasound  MRI  Joint aspiration or synovial biopsy |
| **Blood vessels** | Arterial aneurysms, arterial or venous thrombosis, including thromboembolic disease, etc. | Normal physical examination | Doppler ultrasound  CT angiography  MR angiography |
| **Other** | Endometritis, mastitis, ophthalmologic involvement, etc. | Normal physical examination | Adapt according to the context |
| * Cardiac MRI is only warranted in case of abnormal ECG, troponin, NT-proBNP and/or TTE findings. Additionally, it may also be considered as a first-line test in clonal HES due to the increased incidence of cardiac involvement, especially when *FIP1L1-PDGFRA* gene fusion is evidenced. Coronary angiography or coronary artery spasm test based on the clinical setting.  ** While some invasive procedures are necessary to confirm HES-related organ involvement and rule out differential diagnoses, their indications should be discussed on a case-by-case basis. | | | |
